# Clinical surveillance systems obscure the true cholera infection burden in an endemic region

**DOI:** 10.1038/s41591-024-02810-4

**Published:** 2024-02-20

**Authors:** Sonia T. Hegde, Ashraful Islam Khan, Javier Perez-Saez, Ishtiakul Islam Khan, Juan Dent Hulse, Md Taufiqul Islam, Zahid Hasan Khan, Shakeel Ahmed, Taner Bertuna, Mamunur Rashid, Rumana Rashid, Md Zakir Hossain, Tahmina Shirin, Kirsten E. Wiens, Emily S. Gurley, Taufiqur Rahman Bhuiyan, Firdausi Qadri, Andrew S. Azman

**Affiliations:** 1 Department of Epidemiology, Johns Hopkins Bloomberg School of Public Health, Baltimore, MA USA; 2 Infectious Disease Division, icddr,b (International Centre for Diarrhoeal Disease Research, Bangladesh), Dhaka, Bangladesh; 3 Unit of Population Epidemiology, Geneva University Hospitals, Geneva, Switzerland; 4 Geneva Centre for Emerging Viral Diseases, Geneva University Hospitals, Geneva, Switzerland; 5 Bangladesh Institute of Tropical and Infectious Diseases, Chattogram, Bangladesh; 6 Institute of Epidemiology, Disease Control and Research, Dhaka, Bangladesh; 7 Department of Epidemiology, Temple University, Philadelphia, PA USA; 8 Division of Tropical and Humanitarian Medicine, Geneva University Hospitals, Geneva, Switzerland

**Keywords:** Bacterial infection, Epidemiology

## Abstract

Our understanding of cholera transmission and burden largely relies on clinic-based surveillance, which can obscure trends, bias burden estimates and limit the impact of targeted cholera-prevention measures. Serological surveillance provides a complementary approach to monitoring infections, although the link between serologically derived infections and medically attended disease incidence—shaped by immunological, behavioral and clinical factors—remains poorly understood. We unravel this cascade in a cholera-endemic Bangladeshi community by integrating clinic-based surveillance, healthcare-seeking and longitudinal serological data through statistical modeling. Combining the serological trajectories with a reconstructed incidence timeline of symptomatic cholera, we estimated an annual *Vibrio cholerae* O1 infection incidence rate of 535 per 1,000 population (95% credible interval 514–556), with incidence increasing by age group. Clinic-based surveillance alone underestimated the number of infections and reported cases were not consistently correlated with infection timing. Of the infections, 4 in 3,280 resulted in symptoms, only 1 of which was reported through the surveillance system. These results impart insights into cholera transmission dynamics and burden in the epicenter of the seventh cholera pandemic, where >50% of our study population had an annual *V. cholerae* O1 infection, and emphasize the potential for a biased view of disease burden and infection risk when depending solely on clinical surveillance data.

## Abstract (web summary)

Integrated analysis of clinical, serological and behavioral data estimates that current clinical surveillance systems detect only 1 of 3,280 true community infections in a cholera-endemic region of Bangladesh.

## Main

More than half a century into the seventh cholera pandemic, *V. cholerae* El Tor O1 continues to ravage communities lacking access to safe water and sanitation. In contrast to the few official cholera cases reported to the World Health Organization (WHO) each year from South Asia, this region probably bears a substantial portion of the global burden of cholera and serves as the source of genetic diversity within the seventh pandemic lineage^1–3^. Although past surveillance efforts have provided glimpses into the burden of cholera in South Asia, their scope has been limited due to nonexhaustive testing of acute watery diarrhea cases (suspected cholera), which could result from a number of other related pathogens and a focus on only medically attended cases^4–7^. Viewing cholera through the lens of passive surveillance systems alone may lead to severe underestimates of incidence and a skewed understanding of the transmission dynamics and population immunity related to pandemic *V. cholerae* O1. This understanding is critical for designing tailored interventions to curb transmission and reduce the burden of disease, especially in South Asia, the posited epicenter of cholera.

Historical estimates of cholera seroincidence from South Asia have revealed a wide gap between infections—defined by an immunological boost of *V. cholerae* O1-specific antibodies with or without clinical disease—and the number of cholera cases detected through clinic-based surveillance, ranging from close to 1 case per infection to well over 100 (refs. ^8–11^). Analyses from a national serosurvey in Bangladesh suggested that 1 in 5 people has at least one infection each year with *V. cholerae* O1, yet fewer than 1,200 cholera cases were reported to the WHO annually over the past decade^12^^,^^13^ (www.who.int/data/gho). Although both serologically inferred infections and clinical cases provide important clues about the true burden of disease, each data type has its own limitations and biases, and methods to integrate both types of data and translate one derived metric to another (that is, infections to medically attended cases and vice versa) are lacking.

**Fig. 1:**
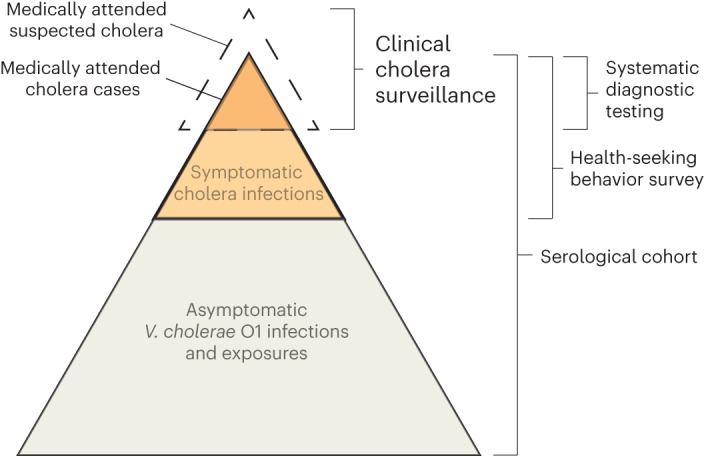
Overview of conceptual model of the continuum between infections and medically attended cholera. Key data sources used in the model are indicated on the right. The dashed area indicated by ‘medically attended suspected cholera’ represents those suspected cases that are false positive by cholera diagnostic testing and therefore captured by surveillance.

Only a fraction of infections appears as confirmed cases within a facility-based surveillance system, which can be the result of a suite of host and pathogen factors (Fig. 1). Serologically inferred infections represent exposures to *V. cholerae* O1, leading to a boost in antibodies, but only a fraction of these lead to symptomatic disease, which can be explained partially by differences in exposure routes, inoculum size, previous exposures to *V. cholerae* O1 and circulating strains^8^. Furthermore, on experiencing a symptomatic *V. cholerae* O1 infection, only a portion of individuals will seek care at formal medical facilities, depending on disease severity and access to care. Although diarrhea-related care-seeking data for children aged >5 years are abundant, largely through the Demographic and Health Surveys^14,15^, such data for older children and adults are scant, thereby limiting methods for translating estimates of infection into estimates of burden of disease. Passive clinic-based surveillance is most commonly employed for tracking cholera mainly through the symptom-based classification of suspected cholera; however, laboratory confirmation is rare and the suspected cholera case definition is known to be highly nonspecific^16^. A systematic review suggested that, on average, less than half of clinically suspected cholera cases captured by surveillance are true *V. cholerae* O1 infections, but this proportion varied widely over time and space^17^. Understanding the pathway from infections with *V. cholerae* O1 to confirmed and reported clinical cholera is crucial for interpreting passive clinical surveillance data and serosurveys, making inferences about transmission dynamics and disease burden and ultimately improving our ability to target resources in the fight against cholera.

In the present study, we combine data from a longitudinal serological cohort, enhanced clinical and laboratory surveillance and a health-seeking behavior survey to elucidate the pathway between exposure to *V. cholerae* O1 and clinical disease, and estimate the true incidence of infections and symptomatic cholera disease in an endemic subdistrict of Bangladesh^7,12^.

## Results

### Medically attended suspected cholera cases

**Extended Data Fig. 1:**
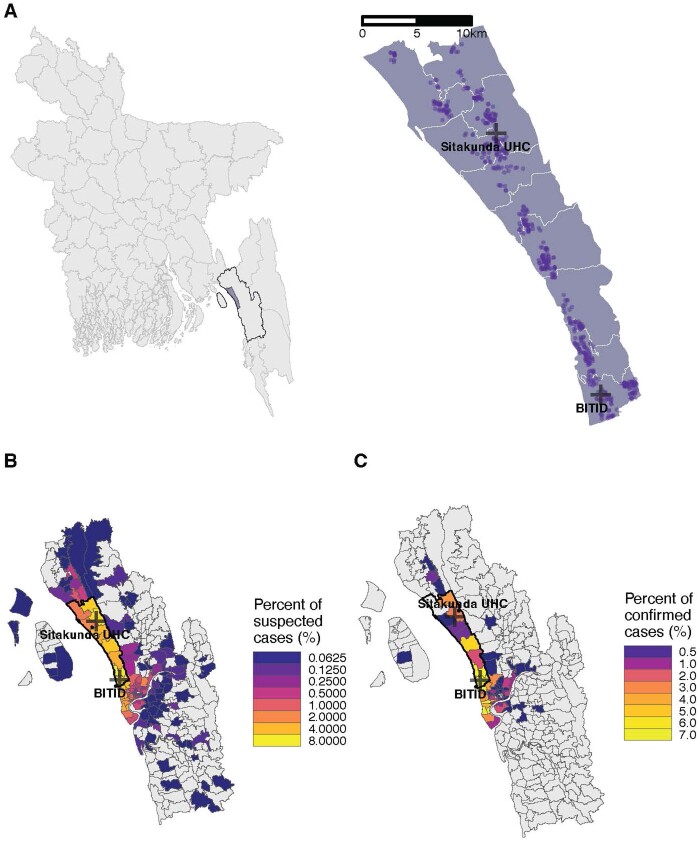
Map of the study area. Map of the enrolled households in the serosurvey in the Sitakunda sub-district (**a**), and the suspected (**b**) and confirmed (**c**) clinical *V. cholerae* cases by RDT test result by Union (the lowest administrative unit) in the Chattogram district of Bangladesh. Among the 2,176 suspected cases that visited the study clinical facilities during the study period, 99% (N = 2,158) were from or spent the last 7 days in the Chattogram district. Among the suspected cases from the Chattogram district (N = 2,158), 46% (N = 998) were from or spent the last 7 days in the subdistrict of Sitakunda.

Through enhanced clinical surveillance from 24 January 2021 to 13 February 2022, we detected 2,176 suspected cholera cases (defined as those aged ≥1 years experiencing three or more watery, nonbloody stools in the last 24 h) at the two primary sites treating diarrhea cases in the Sitakunda subdistrict (population size ∼450,095 for those aged ≥1 years), the geographical focus of our analyses (Extended Data Fig. 1). Of the suspected cases, 34% (745 of 2,176) were aged <5 years and 46% (998 of 2,176) were from or spent the last 7 d in Sitakunda. Nearly all (99.6%) of the suspected cases from Sitakunda were admitted to the health facility and experienced some (96%) or severe (3%) dehydration (Table 1); there were no reported deaths. The annual incidence rate of suspected cholera cases in Sitakunda alone was 2.1 per 1,000, including 10.5 per 1,000 among those aged 1–4 years, 1.2 per 1,000 among those aged 5–64 years and 1.9 per 1,000 among those aged ≥65 years.

**Table 1:** Overview of suspected cases by RDT status for participants enrolled into clinical surveillance from 24 January 2021 to 13 February 2022, the end of the last serosurvey, who resided in the Sitakunda subdistrict (*n* = 998)

Characteristic	RDT-negative *n* = 912	RDT-positive *n* = 86
**Site,** ***n*** **(%)**^a^		
BITID	488 (54)	67 (78)
Sitakunda Upazila Health Complex	424 (46)	19 (22)
**Sex,** ***n*** **(%)**		
Male	481 (53)	48 (56)
Female	431 (47)	38 (44)
Age, median (IQR)^a^	5 (1–35)	25 (14–45)
**Age group,** ***n*** **(%)**^a^		
1–4 years	450 (49)	16 (19)
5–64 years	432 (47)	63 (73)
65+ years	30 (3.3)	7 (8.1)
**Patient type,** ***n*** **(%)**		
Inpatient	907 (99.5)	86 (100)
Outpatient	5 (0.5)	0 (0)
**Dehydration status**		
None	5 (0.5)	0 (0)
Some	892 (98)	71 (83)
Severe	15 (1.6)	15 (17)
**Culture,** ***n*** **(%)**^b^		
Negative	0 (0)	36 (42)
Positive	0 (0)	50 (58)
Not tested	912	0
**PCR,** ***n*** **(%)**^a,c^		
Negative	388 (93)	25 (29)
Positive	30 (7.2)	61 (71)
Not tested	494	0
Self-reported antibiotic use 48 h before admission, *n* (%)^a^	750 (82)	54 (63)
No. of days between symptom onset and seeking care, mean (s.d.)^a^	1.44 (1.10)	1.08 (1.06)
No. of days between healthcare facility admission and discharge, mean (s.d.)	1.64 (2.08)	2.06 (5.48)

^a^Significant difference by Wilcoxon’s rank-sum test or Pearson’s *χ*^2^ test; IQR, interquartile range.

^b^Confirmation testing by culture was performed only on RDT-positive samples.

^**c**^Confirmation testing by PCR was performed only on RDT-positive samples and a subset of RDT-negative samples.

### Medically attended true cholera cases

**Fig. 2:**
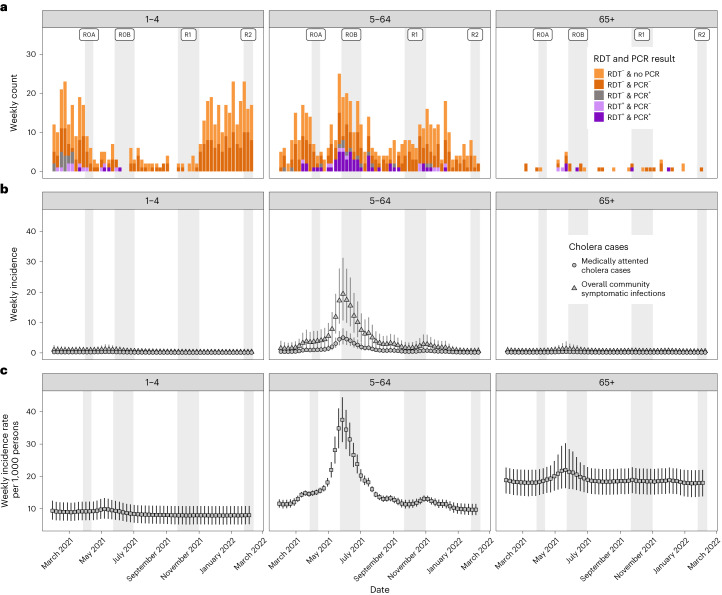
Weekly suspected cases, symptomatic infections and exposures/infections by age group. **a**, The weekly number of suspected cases from Sitakunda colored by RDT and PCR results (culture results not shown). All suspected cases (*n* = 998) were tested by RDT but only positives and around half of the negatives were tested by PCR. **b**, The weekly estimates of the number of true cholera cases seeking care at study facilities (circles), with triangles representing the estimates of the true number of symptomatic cases both in facilities and in the community. **c**, Representation of estimates of the number of weekly infections that elicit an immunological boost, as inferred from longitudinal vibriocidal titers. Uncertainty is represented by vertical error bars across **b** and **c** giving the 95% CrIs from 5,000 HMC posterior draws. Here, RDT refers to the Cholkit rapid diagnostic test, R0A and R0B refer to the baseline round of the serosurvey from 27 March 2021 to 13 June 2021, which included an ∼1-month gap owing to a national COVID-19-related lockdown (R0A refers to pre-lockdown and R0B to post-lockdown); R1 refers to the first follow-up serosurvey from 21 September 2021 to 9 October 2021 and R2 to the second follow-up serosurvey from 25 January 2022 to 13 February 2022.

As the suspected case definition is known to have low specificity for infections with *V. cholerae* O1 (ref. ^16^), we performed rapid diagnostic tests (RDTs) on all suspected cholera cases, PCR and culture on all RDT-positive tests and PCR on roughly half of all RDT-negative tests. After taking into account the performance of the array of diagnostic tests used (Table 1), we reconstructed the true weekly incidence of medically attended cholera cases (that is, those true cases that sought care at the study surveillance sites), which displayed a distinct temporal signature with most cases (54%) confined to just a 4-month period (April to July 2021; Fig. 2). The incidence rate of medically attended cholera (0.2 per 1,000 per year; 95% credible interval (CrI) = 0.1–0.2) was roughly one-tenth of the observed suspected cholera incidence rate, with children aged 1–4 and those aged >65 years having 2–5× the medically attended incidence rates of the others (Table 2). This implies that, for every 10 (95% CrI 7–10) suspected cholera cases at facilities in Sitakunda, one of these is caused by *V. cholerae* O1, with strong temporal and age-specific heterogeneity, probably due to the incidence of other seasonal pathogens (Supplementary Table 1)^18,19^.

**Table 2:** Estimates of annualized incidence rates per 1,000 population

	≥1–4 years	5–64 years	>65 years	Overall
**Healthcare facility incidence rates (per 1,000 population)**				
Suspected cholera incidence	10.5	1.2	1.9	2.1
RDT-positive incidence	0.4	0.2	0.4	0.2
Medically attended cholera incidence	0.2 (0.1–0.3)	0.1 (0.1–0.2)	0.5 (0.3–0.8)	0.2 (0.1–0.2)
**Community incidence rates (per 1,000 population)**				
Symptomatic cholera incidence (community + clinics)	0.7 (0.4–1.2)	0.6 (0.4–0.7)	2.0 (1.0–3.4)	0.6 (0.5–0.8)
Incidence of infections (community)	325.8 (234.5–414.6)	539.4 (518.1–562.1)	602.5 (512.8–686.1)	534.6 (514.3–555.7)

The uncertainty for some estimates, in parentheses, is derived using the 95% CrIs from 5,000 HMC posterior draws.

### Health-seeking behavior

**Fig. 3:**
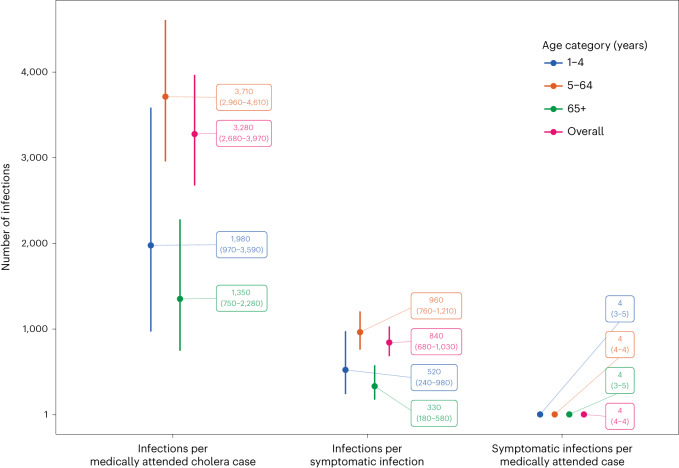
Estimates of ratios along the continuum between infections and reported medically attended cholera cases by age group (color). Uncertainty is represented by vertical error bars giving the 95% CrIs from 5,000 HMC posterior draws, whereas the center gives the mean of the posterior draws. These ratios are derived from the three primary data sources used: clinic-based surveillance (*n* = 998), the healthcare-seeking survey (*n* = 2,481) and the serological cohort (*n* = 1,785).

**Extended Data Fig. 2:**
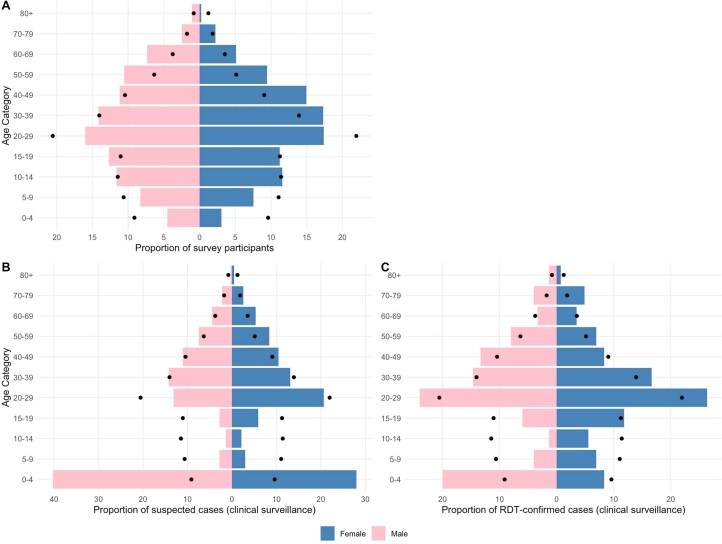
Demographic pyramid of survey participants. Demographic pyramid of survey participants (only those with three follow-up visits) (**a**) and of suspected (**b**) and RDT-confirmed (**c**) cases from clinical surveillance by age group and sex. The dots represent the underlying population distribution. The enrolled suspected and confirmed cholera cases in clinical surveillance roughly followed the age and sex distribution of the national population, though children less than five years old were overrepresented when compared to the population and those 5–19 years old were underrepresented.

Not all people will seek healthcare for diarrhea and, among those who do, only a fraction will report to facilities that are part of official disease surveillance systems^20^. To understand care-seeking behaviors for cholera-like symptoms, we conducted a survey of 2,481 individuals from 580 households (Methods), representing the catchment area of clinical surveillance, and asked each person about their healthcare-seeking preferences. The survey included 53% females and had a similar demographic profile to the national population with notable undersampling of young children (Table 3 and Extended Data Fig. 2). When asked whether they would seek any care for acute watery diarrhea with some dehydration, 86% (*n* = 2,128) of participants indicated that they would, with the likelihood decreasing by age (Supplementary Table 2). Almost half (49%) said that they would seek care at a pharmacy and 26% that they would seek care at one of the two official clinical surveillance facilities for diarrhea in Sitakunda, and the distribution of care seeking by age did not differ between healthcare facility type (Supplementary Table 3). Overall, we estimate that, for every 4 (95% CrI = 3.7–4.1) infected individuals with moderate-to-severe diarrhea, only 1 would seek care at the official cholera surveillance facilities in Sitakunda and be captured as a suspected cholera case, with no significant differences by age (Fig. 3).

**Table 3:** Overview of the participants enrolled into the serosurvey

Characteristic	Serological cohort *n* = 1,785	Survey data *n* = 2,481	*P* value^a^
**Sex,** ***n*** **(%)**			0.81
Male	842 (47)	1,161 (47)	
Female	943 (53)	1,320 (53)	
Age, median (IQR)	28 (15–45)	28 (15–45)	0.97
**Age group,** ***n*** **(%)**			0.35
1–4 years	67 (3.8)	111 (4.5)	
5–64 years	1,625 (91)	2,222 (89.5)	
65+ years	93 (5.2)	148 (6.0)	
Would seek medical care if experiencing 3+ loose stools in a day, *n* (%)^b^	1,349 (76)	1,873 (76)	0.94
Would seek medical care if experiencing 3+ loose stools in a day and dehydration, *n* (%)	1,514 (85)	2,128 (86)	0.38
Would seek medical care if experiencing 3+ loose stools in a day for >3 d, *n* (%)	1,678 (94)	2,327 (94)	0.77
**Highest educational attainment,** ***n*** **(%)**			0.68
No schooling	268 (15)	416 (17)	
Primary	548 (31)	773 (31)	
Lower secondary	546 (31)	730 (29)	
Upper secondary	325 (18)	433 (17)	
Bachelor degree	76 (4.3)	98 (4.0)	
Postgraduate	21 (1.2)	29 (1.2)	
Unknown	1	2	

These data are separated by those who were enrolled and provided serum samples for all three rounds of the serosurvey (*n* = 1,785; serological data) and those who were enrolled and provided survey data during at least one study visit (*n* = 2,481; survey data). There are no statistically significant differences among the two survey populations using the following demographic characteristics; Wilcoxon’s rank-sum tests and Pearson’s *χ*^2^ tests were used to test differences.

^a^Wilcoxon’s rank-sum test; Pearson’s *χ*^2^ test.

^b^One individual had an ‘unknown’ response.

### *V. cholerae* O1 infections

Although *V. cholerae* O1 infections manifest across the spectrum of clinical disease severity, the proportion of infections leading to symptomatic disease is unclear. To understand the incidence of infections, independent of clinical manifestations, we followed participants of the representative health-seeking survey over the course of 1 year and collected three blood draws per person (*n* = 1,785 participated in all three visits; Fig. 1 and Table 3). Of individuals in our serological cohort, 53% were female and most reported using piped water during the high transmission season compared with other times (Supplementary Table 5). We measured vibriocidal titers, an established marker of recent infection, for each sample, following standard methods^21,22^. Given that vibriocidal titers decay within the time between study visits (half-life of 74–201 d (ref. ^23^); time between study visits 87–214 d), we developed a Bayesian approach to reconstruct the unobserved infection histories of each participant in our serological cohort, taking into account both antibody kinetics and measurement error (Methods).

Combining the serological trajectories with the reconstructed incidence of symptomatic cholera, we estimated an annual *V. cholerae* O1 infection incidence rate of 535 per 1,000 population (95% CrI = 514–556), with incidence increasing by age group (Table 2). Those aged 1–4 years had an infection incidence of 326 per 1,000 (95% CrI = 234–415), which was notably lower than other age groups. For every 840 infections (95% CrI = 680–1,030), we estimated that only 1 infection would result in symptomatic disease. Young children and elderly people had a two to three times higher probability of symptomatic disease from infection than the rest of the population, which could potentially be explained by age-dependent exposure routes and infectious dose thresholds, lack of prior immunity among children and immunosenescence among elderly people. The incidence rate of symptomatic cholera, including both those who were medically attended and those who were not, was 0.6 per 1,000 per year (95% CrI = 0.5–0.8) with elderly people having twice the incidence rate compared with others (Table 2). Notably, when care seeking was based on alternative definitions of diarrhea severity, the symptomatic incidence rate estimates did vary (0.4–1.1 per 1,000 per year) (Supplementary Table 6).

### Clinical cholera surveillance versus *V. cholerae* O1 infections

Clinic-based surveillance alone underestimated the number of infections and led to a distorted view of their timing across the surveillance period. Our estimates imply that, for every 3,280 (95% CrI = 2,680–3,970) infections in the community, only 1 ended up as a medically attended cholera case captured by the surveillance system and, if cholera confirmation was done with traditional microbiological culture, this gap would grow even wider owing to imperfect sensitivity (4,551 infections per medically attended cholera case; 95% CrI = 3,677–5,591).

The relationship between infections in the community and observed clinical cholera cases was not constant over time. We initially conceptualized that the rates of clinical cholera and infections were proportional throughout the study period in our Bayesian model of infection incidence. However, we found strong statistical support (leave-one-out cross-validation–Pareto smoothed importance-sampling (LOO-PSIS) difference of 78.7, s.e.m. = 19.0) for an alternative model where seroincidence rates were composed of two components: one proportional to clinical cholera incidence rates and the other a constant hazard throughout the period independent of observed clinical cases, which was ultimately used in our primary analyses. This model formation, which served as the basis for our primary results, suggests that, even at times when no medically attended cholera cases happen, >4,000 infections (weekly range: 4,546–5,960, annualized weekly incidence rate 11.4–15.0 per 1,000 people per year) occur each week in Sitakunda.

## Discussion

Through joint inference on data from enhanced clinical and laboratory surveillance, a longitudinal serological cohort and a healthcare-seeking behavior survey, we illustrate a landscape of regular immunological exposures to *V. cholerae* O1 where resulting cholera disease is the exception rather than the rule. We estimated that, for every 3,280 infections, 4 will result in disease and, of these, only 1 will seek care and ultimately be captured by the surveillance system. Passive clinic-based surveillance, the lens through which cholera is typically viewed in most of the world, captures only a fraction of symptomatic cases and obscures our understanding of the dynamics and magnitude of *V. cholerae* O1 infections. Our results shed light on the continuum between infections and medically attended disease in endemic populations and provide estimates of the true burden of cholera.

Although several investigators and decision-making bodies have attempted to estimate the true burden of symptomatic cholera, they have primarily used facility-based suspected case data, often without laboratory confirmation. Most studies have not adjusted for health-seeking behaviors, diagnostic test performance (when used) or severity of infection, possibly resulting in biased estimates of risk and poor allocation of resources^24,25^. Perhaps the most widely used estimate of cholera incidence in Bangladesh, for several policy and planning documents, was based on an extrapolation of medically attended cholera incidence measured in 2005 in the neighboring area of Kolkata, India (∼1.64 per 1,000 people)^24^. Another study conducted in 6 sentinel sites around Bangladesh from 2010 to 2011, which attempted to estimate the incidence of severe cholera by using hospital admissions data and a care-seeking survey, found that incidence rates ranged across sites from 0.3 to 4.9 per 1,000 per year and care-seeking probabilities were consistent with those estimated in our study^25^. Unlike previous studies, we incorporated serologically derived incidence data and accounted for diagnostic test performance in our estimation, approximating the true burden of cholera in this endemic setting to be 0.6 symptomatic infections per 1,000 per year. Although our estimate falls within the lower range of historical estimates, we cannot reliably attribute differences to true changes in incidence given the prior use of alternative, less sensitive methods. Although it is unclear whether our estimated ratio of infections to symptomatic disease is generalizable to other parts of Bangladesh, combining this ratio with the national seroincidence results from a 2015–2016 serosurvey^12^ would imply that roughly 34,500 symptomatic cholera cases occurred nationally in the year before this survey.

Our approach allowed us to estimate and partition the overall incidence of *V. cholerae* O1 infections, those that result in symptoms and eventually care seeking and those that remain subclinical (Fig. 1). Far greater than previous estimates from cross-sectional serological data alone (20%, 95% CrI = 10.4–35.2, for all the Chattogram District^12^), we estimated that at least 50% of the Sitakunda population are infected with *V. cholerae* O1 annually and that >99% of these infections are subclinical (Supplementary Table 4). Such a high level of infection incidence has been documented only in outbreaks in previously naive populations, for example, just after the initial appearance of cholera in Haiti in 2010 (ref. ^26^), and thus has been attributed to a lack of pre-existing immunity. The high proportion of subclinical infections observed in Sitakunda could, however, represent transient infections or exposures that do not result in gut colonization or bacterial shedding and/or asymptomatic or less severe infections, all possibly influenced by previously acquired immunity. Data from human challenge studies among North American volunteers suggest that only a small proportion of exposures that don’t lead to shedding or clinical disease mount a robust vibriocidal response, but this may not generalize to populations with endemic cholera^27^. Both plausible explanations have implications for our understanding of endemic cholera transmission dynamics. The observed high incidence rate in Sitakunda, combined with our understanding of the reproductive number of cholera (typical range one to three^28–31^) and the biannual seasonal cholera peaks^7^, is consistent with the hypothesis that not all infections lead to the same magnitude and duration of protection against clinical disease. Previous modeling work implied that mild and asymptomatic infections^32^ or inadequate levels of crossprotection between serotypes^33^ may lead to faster immune waning, resulting in endemic areas bearing a consistently high infection incidence, but strong empirical evidence is still lacking. Despite the apparent disconnection between infection and subsequent protection, which may be explained by heterogeneity in inoculum size, immune responses and/or infection histories, our study highlights potential hypotheses and corroborates these as targets for future investigations.

**Extended Data Fig. 3:**
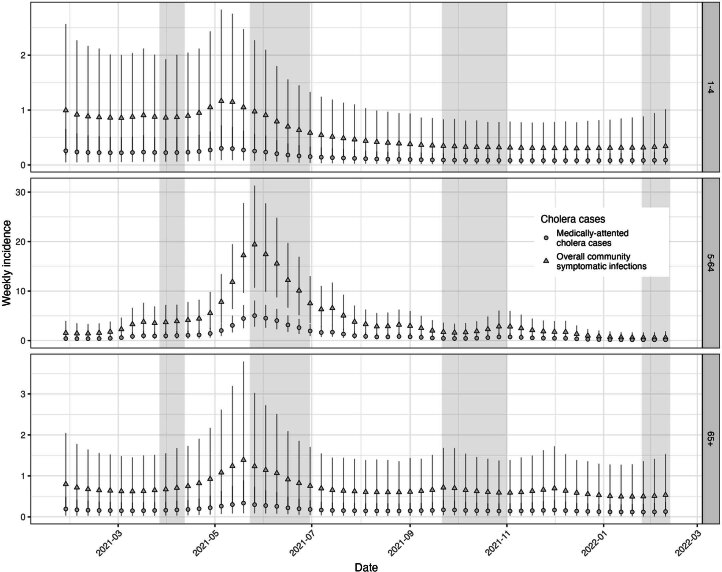
Weekly estimates of the number of true cholera cases seeking care at the study health facilities. The weekly estimates by age group of the number of true cholera cases seeking care at study facilities (circles) with triangles representing the estimates of the true number of symptomatic cases both in facilities and the community. Uncertainty is represented by vertical error bars across the middle and bottom rows giving the 95% credible intervals from 5,000 Hamiltonian Monte Carlo (HMC) posterior draws, while the center gives the mean value of the posterior draws.

By inferring time-varying infections, our results contribute to our understanding of the different pathways through which cholera transmission occurs. Two views on the dominant routes of *V. cholerae* infections leading to clinical disease have emerged over the past 30 years: the first suggests that infections occur through exposure to *V. cholerae* in the aquatic environment and the second through more proximal exposures to infected individuals, often via food and/or water^34,35^. We found statistical support that infections in Sitakunda could be explained by two components: one linked to the number of cases observed at health facilities and the other a constant hazard from an unknown source. This finding may be consistent with the dual roles of frequent low-dose exposures to environmental *V. cholerae* (O1 and other serogroups) and seasonal exposures to higher doses of bacteria, potentially of the hyper-infectious phenotype^36^, from symptomatic individuals. As the Bay of Bengal forms the entire western border of Sitakunda, frequent exposures to aquatic flora and fauna, which have been associated with *V. cholerae* infection, is plausible^37^. A well-documented dose–response relationship exists because higher doses of *V. cholerae* O1 bacteria lead to more severe disease^38^, and this high-dose exposure pathway may be seasonal in this endemic population. The temporal clustering of symptomatic disease (Extended Data Fig. 3) may furthermore be a result of seasonal phage predation^39^ or changing routes and/or manners of bacterial ingestion^40^, which could be age dependent. Future detailed models exploring the forces behind these apparent different drivers of infection could ultimately improve our understanding of *V. cholerae* O1 dynamics.

Our results not only have implications for our understanding of the dynamics of this ancient disease, but may provide important practical insights for the global fight against cholera and the End Cholera 2030 roadmap^41^. Several countries have devised national cholera control plans that rely on passive clinical surveillance and have bold targets, often including elimination of the disease by 2030. Our results affirm previous modeling results that inapparent infections may be key to understanding *V. cholerae* O1 transmission in endemic areas^32^. This implies that the absence of cases may not mean absence of transmission, which must be considered when approaching elimination. After 3 years of no confirmed cases in Haiti, a new outbreak emerged in 2022, and genomic evidence linked the bacterium to strains circulating in the country in 2010. Cryptic transmission from subclinical infections (in the face of waning immunity and a lack of clean water and sanitation) and an environmental reservoir have both been proposed as plausible explanations^42^. Therefore, the factors shaping this potential disconnection are probably critical for designing interventions for elimination, such as vaccines, water and sanitation, assessing risk and establishing appropriate surveillance systems.

Although the most widely used cholera vaccines (killed whole oral vaccines) protect against severe disease, it is unclear how or if they protect against subclinical infection, particularly those that may lead to onward transmission^43^. Defining the immunological markers of subclinical infections, correlates of protection and the resulting different endpoints for clinical studies can help us further understand the mechanisms of current vaccines and lead to the development of better ones in the years to come. Furthermore, when exposures and risk are this pervasive, our results also strengthen the argument for safe, well-managed water systems. Individuals in our serological cohort reported using piped water more often during the high transmission season compared with other times (Supplementary Table 5), suggesting a lack of consistent service and/or contamination^44,45^. Although few cholera studies have estimated such a high proportion of subclinical infections, a high infection to medically attended case ratio (16–32 infections per symptomatic case) has been documented for typhoidal salmonellae, another enteric infection, among Bangladeshi children^46^. These concordant results could provide the impetus for aligned, crosspathogen, horizontal intervention mechanisms, specifically for clean water and sanitation.

Our study comes with several limitations. First, we based our model on the most influential marker of recent infection to estimate seroincidence, vibriocidal titers, although other antibody responses may contain additional information to help improve seroincidence estimates^47^. We focused on vibriocidal titers given the existence of a well-characterized postinfection kinetic model from medically attended cholera cases calibrated mostly on adults^23^. Future efforts may attempt to model the simultaneous decay of multiple markers ideally based on validation sets that include both mild and severe cholera infections. Second, our representative health-seeking behavior survey results suggested that roughly one in four people with symptomatic cholera would seek care at one of the primary surveillance sites in Sitakunda for cholera-like symptoms. This assumed that responses related to hypothetical moderate diarrhea represented the ‘average’ severity of disease; however, estimates of symptomatic incidence rates did vary when care seeking was based on alternative diarrhea severity definitions (Supplementary Table 6). It is likely that the clinical spectrum of disease varies across age groups (for example, elderly people and children are more likely to have severe disease) and defining this distribution can aid in future work where these assumptions are needed. Furthermore, the hypothetical care-seeking questions that we used in this survey may not capture the true behaviors of participants, probably overestimating people’s propensity to seek care. Larger studies asking about recent diarrheal events and subsequent care seeking could help improve our understanding of care seeking for cholera. Third, the observed clinical incidence is based on data collected from only two healthcare facilities and not all the locations where individuals with cholera may seek care, such as pharmacies or private clinics. As the distribution of care seeking by age did not differ much between healthcare facility types (Supplementary Table 3), we assumed that the age-specific and temporal trends in our observed facilities reflected those of other healthcare facilities in the catchment area. Fourth, our inference in the present study is based on data gathered from 1 year, 2021–2022, and in a single epidemiological setting. The generalizability of these results temporally and geographically, therefore, remains unclear and warrants future studies in other endemic settings across South Asia and the African continent, where the epidemiology of cholera probably differs^48^. Even so, these data and results illustrate the importance of integrated disease surveillance for understanding cholera transmission and risk.

Through combining serological, epidemiological and behavioral data, we deconstructed the continuum between *V. cholerae* O1 infections and medically attended cholera cases observed within a typical surveillance system in an endemic community in Bangladesh. We revealed far higher than previously described rates of infection incidence with more than half the population infected each year and 4,551 infections per observed culture-positive, medically attended cholera case. The methods that we developed in the present study allowed us to map out a larger framework of burden estimation and scale the number of infection events in the community to symptomatic cases, medically attended cases and test-positive, medically attended cases that are ultimately counted within surveillance networks. These data support the relevance of quantifying subclinical infections in endemic settings, so that we can better understand the natural history of *V. cholerae* O1 infection and adequately develop and allocate resources at the population level to eliminate disease. Gaining a deeper understanding of subclinical infections in Bangladesh, where six of the last seven pandemics began, and establishing surveillance systems that extend beyond ‘official’ healthcare facilities, which capture only a small fraction of true incidence, may be key to making serious progress in the global targets for cholera control.

## Methods

### Clinical surveillance of suspected cholera cases

Our study took place in the Sitakunda subdistrict (population size ∼450,095 for those aged ≥1 year) located in the Chattogram district of Bangladesh. Two public health facilities provided care for patients wiith diarrhea: the Sitakunda subdistrict hospital (Upazila Health Complex), located toward the north of the subdistrict, and the Bangladesh Institute for Tropical Infectious Diseases (BITID), located toward the southern end of the subdistrict, near the city of Chattogram. These two sites are the primary sites for surveillance of diarrheal disease and cholera for the Bangladesh Directorate General for Health Services in Sitakunda. From 24 January 2021 to 13 February 2022, we undertook surveillance of the inpatient and outpatient wards of both health facilities and attempted to enroll all suspected cases aged ≥1 year presenting with nonbloody, acute watery diarrhea (three or more loose stools in the 24 h preceding the visit). After obtaining informed consent, we administered a short structured questionnaire using the RedCap software and collected a stool (or rectal swab) specimen for laboratory analyses. We tested each patient’s fecal sample onsite with the Cholkit RDT (Incepta), then placed the sample in Cary Blair medium and on Whatman 903 filter paper for subsequent lab testing at icddr,b in Dhaka. Owing to the expected high sensitivity and moderate specificity of the RDT^49^, all RDT-positive samples were tested by culture and end-point PCR (for *ctxA* and *rfb* genes from filter paper as described in Hosino et al.^50^). Using the same PCR protocol, we additionally tested a random subset of nearly half of the RDT-negative samples (46%). All diagnostic test results were used in a latent class model to determine the weekly number of confirmed cases as described below.

### Serological cohort and care-seeking survey

We enrolled a population representative cohort in Sitakunda between 27 March 2021 and 13 June 2021, including an ∼1-month gap due to a national COVID-19-related lockdown (referred to as rounds R0A and R0B throughout). To enroll households, we used two-stage sampling based on satellite imagery (Airbus, Pléiades 1B sensor) with digitized building footprints classified as single or multi-story units, where we first divided the Sitakunda subdistrict into 1-km^2^ grid cells and then randomly selected grid cells proportional to the number of households in each with replacement. Within each selected grid cell, we randomly selected structures (or GPS (Global Positioning System) coordinates) weighted by whether they were classified as single- or multi-story units; the number of structures selected by grid cell varied and only one structure was enrolled per GPS coordinate selected. If no structure was found at the point or the structure located at the point was not residential, the study team attempted to enroll the nearest residential household within 20 m or proceeded to the next assigned point if no residential household existed within 20 m. If the structure found was multi-story, the study team enumerated the number of residential households inside and generated a random number to determine which household to attempt to enroll. If no one was at the household once it was found, the study team attempted to revisit up to three times in the following 24 h. If no one was at home after the attempted revisits or the household refused to participate, the study team proceeded to the next point (or attempted to enroll the next closest residence to the right if in a multi-story unit).

After receiving verbal consent from the head of household (or representative), we attempted to enroll all people aged ≥1 year who were members of the household (that is, those who regularly sleep in the household and eat there) and asked for written consent (and assent for those aged 7–17 years) from each person. When household members who met this inclusion criterion were not present, we attempted to revisit up to three times in the survey period to enroll them. Survey staff administered a structured household-level questionnaire to the head (or representative) of each enrolled household. They also administered an individual-level questionnaire to and collected venous blood from each consenting participant (∼5 ml from adults and ∼3 ml from children aged <5 years). The baseline individual-level questionnaire included questions on each person’s demographics, mobility patterns, health status and healthcare-seeking behaviors. Each enrolled household had two follow-up visits at approximately 4-month intervals (R1 from 21 September 2021 to 9 October 2021 and R2 from 25 January 2022 to 13 February 2022). At each follow-up visit, members of the households who were not previously enrolled (or declined to participate in the previous round) were eligible to participate, and all consenting participants were administered a follow-up individual-level questionnaire and venous blood draw, and household heads a follow-up household-level questionnaire. Follow-up questionnaires were aimed at recent clinical disease episodes, healthcare-seeking and water-, sanitation- and hygiene-related behaviors such as individual drinking water sources, household sanitation and water infrastructure.

To gauge people’s propensity to seek care for cholera, we asked a series of questions at baseline of each participant about the three levels of hypothetical diarrhea severity: mild, moderate and severe. We asked whether individuals would seek healthcare for each syndrome:

- Mild diarrhea: after experiencing three or more loose stools in 1 d, would you seek medical care?
- Moderate diarrhea: after experiencing three or more loose stools in a day and dehydration, would you seek medical care?
- Severe diarrhea: after experiencing three or more loose stools per day for more than 3 d, would you seek medical care?

If a participant answered yes, they were then asked about the specific type of care or facility (public, private, pharmacy, traditional healer, and so on) that they would first visit. Although the clinical spectrum of cholera is variable, we focused our main analyses on the self-reported care seeking for moderate diarrhea (experiencing three or more loose stools in a day and dehydration) while exploring the other severities in sensitivity analyses.

Blood samples were centrifuged the day of collection and frozen at −80 °C until the time of analysis (1–11 months). Following previously described methods^22^, we tested each sample with the vibriocidal assay, with all samples from the same participant run on the same plate. Serum samples collected at different rounds were tested for vibriocidal antibody response against the homologous serotype of bacteria using the *V. cholerae* O1 El Tor Ogawa (strain 25049) or Inaba (strain 19479) strains as the target organism^51^. Guinea-pig complement (Sigma-Aldrich) was used at a 1:10 dilution. The optical densities (ODs) of the plates were measured at 595 nm. A known pooled serum (prepared from convalescent sera of 20–30 cholera patients) was used in each plate as a control. The vibriocidal titer was defined as the reciprocal of the highest serum dilutions causing a >50% reduction of the OD at 595 nm when compared with the OD of the control wells without serum. Wells containing physiological saline and growth medium were included on each plate to exclude the possibility of bacterial contamination of the reagents.

### Sitakunda population estimates

We used the reported population size by 5-year age bins for Sitakunda from the 2011 National Census^52^. We extrapolated population counts by age group and sex assuming 1.5% annual population growth for the 10 years between 2011 and 2021. As children aged <1 year were excluded in the survey and clinical surveillance, we subtracted 20% of the total population for the 0- to 4-year age group, following the age distribution reported in the US Census Bureau International Database for Bangladesh in 2021 (ref. ^53^).

### Statistical analysis

**Extended Data Fig. 4:**
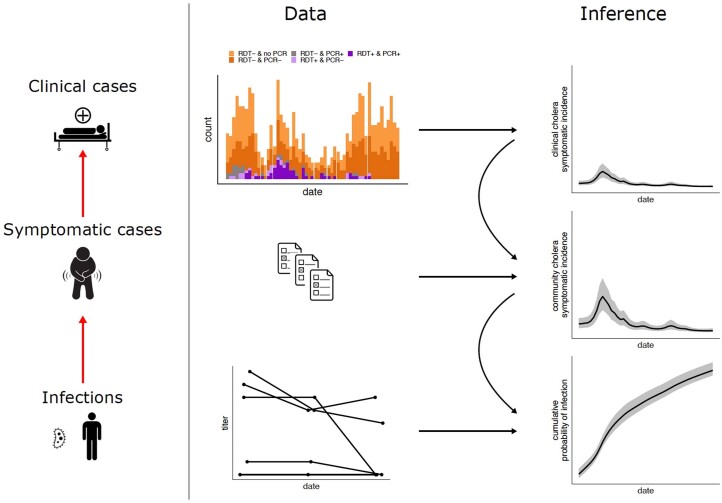
Study aim and the modeling framework. The analysis relies on the inference of seroincidence rates based on serial measurements of vibriocidal antibodies. In turn, this inference relies on estimates of the time-varying force of infection from *Vibrio cholerae* O1, which results in seroconversions in between the study rounds. The icons used in the first column on the left were designed by Freepik (www.freepik.com).

The modeling framework we used consisted of two primary parts: (1) reconstruction of the true time-varying incidence of medically attended cholera from the clinical surveillance data; and (2) modeling of probable titer trajectories in between study visits accounting for infection-induced antibody boosts conditional on a known antibody kinetic model. These two parts are connected through the age-specific time-varying incidence estimates of *V. cholerae* O1, which influences the probability of seroconversion between study rounds (that is, blood draws) for each participant (Extended Data Fig. 4).

#### Reconstruction of medically attended cholera incidence rates

We estimated the age-stratified incidence of medically attended cholera and non-cholera suspected cases within our study facilities using a Bayesian model fit to data from systematic laboratory testing of all suspected cases. Data were restricted to individuals residing in the Sitakunda subdistrict visiting the healthcare facilities from the start of the study period until 13 February 2022, the end of the last serosurvey. We assumed that the incidence of acute watery diarrhea (AWD) (*β*_awd_) can be deconstructed into the sum of the incidence of cholera (*β*_chol_) and non-cholera (*β*_−chol_) AWD. We further assumed that the incidence of both cholera and non-cholera AWD evolved over time during the study period following a first-order Brownian motion at the daily timescale with initial value $\alpha$:

$${\beta }_{{{\mathrm{chol}}}}\left(t\right)=\exp \left({\alpha }_{{{\mathrm{chol}}}}+{\Delta }_{t}\log \left({\beta }_{{{\mathrm{chol}}}}\right)\right),$$

$${\Delta }_{t}\log \left({\beta }_{{{\mathrm{chol}}}}\right)= \left[\log \left({\beta }_{{{\mathrm{chol}}}}\left(t+1\right)\right)-\log \left({\beta }_{{{\mathrm{chol}}}}\left(t\right)\right)\right] \sim {{{N}}}\left(0,{\sigma }_{{\beta }_{{{\mathrm{chol}}}}}\right),$$

$${\beta }_{-{{\mathrm{chol}}}}\left(t\right)=\exp \left({\alpha }_{-{{\mathrm{chol}}}}+{\Delta }_{t}\log \left({\beta }_{-{{\mathrm{chol}}}}\right)\right),$$

$${\Delta }_{t}\log \left({\beta }_{-{{\mathrm{chol}}}}\right)= \left[\log \left({\beta }_{-{{\mathrm{chol}}}}\left(t+1\right)\right)-\log \left({\beta }_{-{{\mathrm{chol}}}}\left(t\right)\right)\right] \sim {{{N}}}\left(0,{\sigma }_{{\beta }_{-{{\mathrm{chol}}}}}\right),$$

$${\beta }_{{{\mathrm{awd}}}}\left(t\right)={\beta }_{{{\mathrm{chol}}}}\left(t\right)+{\beta }_{-{{\mathrm{chol}}}}\left(t\right),$$

$${{\phi }}\left(t\right)=\frac{{\beta }_{{{\mathrm{chol}}}}\left(t\right)}{{\beta }_{{{\mathrm{awd}}}}\left(t\right)},$$

$${n}_{{{\mathrm{awd}}}}\left(t\right)\sim{{\mathrm{Poisson}}}\left({\beta }_{{{\mathrm{awd}}}}\left(t\right)\right),$$

where *β*_awd_ is the total AWD daily incidence and *ϕ* is the time-varying proportion of AWD incidence owing to cholera. We linked the model to data by assuming that the total number of daily AWD cases followed a Poisson distribution with rate *β*_awd_. This likelihood was complemented by the probability of observing specific cholera test results.

Given that we tested all suspected cases with RDTs, which have imperfect sensitivity and specificity^49^, and in some cases PCR and/or culture, which is also imperfect^54^, we modeled the array of test outcomes using a multinomial likelihood, marginalizing over the potential outcomes of tests not performed while accounting for the conditional dependence between the tests inherent in the study design (that is, testing of PCR/culture based on RDT results) with priors on performance derived from a previous study in Bangladesh^54^. Specifically, we first assumed that all three test results were available for all samples and then extended this to the case where they were not available. To account for different potential outcomes, we modeled the joint weekly result of RDT, PCR and culture as a multinomial distribution:

$$\begin{array}{l}\left[{n}_{\left\{-,-,-\right\}},{n}_{\left\{-,-,+\right\}},{n}_{\left\{-,+,-\right\}},\right.\\\left.{n}_{\left\{-,+,+\right\}},{n}_{\left\{+,-,-\right\}},{n}_{\left\{+,-,+\right\}},{n}_{\left\{+,+,-\right\}},{n}_{\left\{+,+,+\right\}}\right] \sim \\ {{\mathrm{multinomial}}}\left({p}_{\left\{-,-,-\right\}},{p}_{\left\{-,-,+\right\}},{p}_{\left\{-,+,-\right\}},{p}_{\left\{-,+,+\right\}},\right.\\\left.{p}_{\left\{+,-,-\right\}},{p}_{\left\{+,-,+\right\}},{p}_{\left\{+,+,-\right\}},{p}_{\left\{+,+,+\right\}}\right),\end{array}$$

where signs in brackets indicate the result of RDT, PCR and culture, respectively (for example, $\left\{-,+,-\right\}$ indicates a negative RDT, a positive PCR and a negative culture result). Each set of outcome probabilities is a function of the true probability of cholera, $\phi$, among suspected cases (that is, the fraction of total AWD owing to cholera) and the test performances. Assuming that the different test outcomes of a suspected cholera case are independent conditional on the probability of cholera, and that all test outcomes are known, the corresponding probabilities are:

$$\begin{array}{ccc}p\left(-,-,-\right) & = & \left(1-{\theta }_{1}^{+}\right)\left(1-{\theta }_{2}^{+}\right)\left(1-{\theta }_{3}^{+}\right)\phi +{\theta }_{1}^{-}{\theta }_{2}^{-}{\theta }_{3}^{-}\left(1-\phi \right),\\ p\left(-,-,+\right) & = & \left(1-{\theta }_{1}^{+}\right)\left(1-{\theta }_{2}^{+}\right){\theta }_{3}^{+}\phi +{\theta }_{1}^{-}{\theta }_{2}^{-}(1-{\theta }_{3}^{-})\left(1-\phi \right),\\ p\left(-,+,-\right) & = & \left(1-{\theta }_{1}^{+}\right){\theta }_{2}^{+}\left(1-{\theta }_{3}^{+}\right)\phi +{\theta }_{1}^{-}\left(1-{\theta }_{2}^{-}\right){\theta }_{3}^{-}\left(1-\phi \right),\\ p\left(-,+,+\right) & = & \left(1-{\theta }_{1}^{+}\right){\theta }_{2}^{+}{\theta }_{3}^{+}\phi +{\theta }_{1}^{-}\left(1-{\theta }_{2}^{-}\right)\left(1-{\theta }_{3}^{-}\right)\left(1-\phi \right),\\ p\left(+,-,-\right) & = & {\theta }_{1}^{+}\left(1-{\theta }_{2}^{+}\right)\left(1-{\theta }_{3}^{+}\right)\phi +\left(1-{\theta }_{1}^{-}\right){\theta }_{2}^{-}{\theta }_{3}^{-}\left(1-\phi \right),\\ p\left(-,-,+\right) & = & \left(1-{\theta }_{1}^{+}\right)\left(1-{\theta }_{2}^{+}\right){\theta }_{3}^{+}\phi +{\theta }_{1}^{-}{\theta }_{2}^{-}(1-{\theta }_{3}^{-})\left(1-\phi \right),\\ p\left(+,-,+\right) & = & {\theta }_{1}^{+}\left(1-{\theta }_{2}^{+}\right){\theta }_{3}^{+}\phi +(1-{\theta }_{1}^{-}){\theta }_{2}^{-}(1-{\theta }_{3}^{-})\left(1-\phi \right),\\ p\left(+,+,-\right) & = & {\theta }_{1}^{+}{\theta }_{2}^{+}\left(1-{\theta }_{3}^{+}\right)\phi +(1-{\theta }_{1}^{-})\left(1-{\theta }_{2}^{-}\right){\theta }_{3}^{-}\left(1-\phi \right),\\ p\left(+,+,+\right) & = & {\theta }_{1}^{+}{\theta }_{2}^{+}{\theta }_{3}^{+}\phi +(1-{\theta }_{1}^{-})\left(1-{\theta }_{2}^{-}\right)(1-{\theta }_{3}^{-})\left(1-\phi \right),\end{array}$$

where *θ*^+^ and *θ*^−^ are the test sensitivity and specificity, and subscripts 1, 2 and 3 denote RDT, PCR and culture, respectively. As data were not available for all tests and samples, however, the likelihood of the sequence of samples could not be expressed as the product of multinomial probabilities described above. Instead, the testing results in our data were one of three cases: (1) only RTD-negative results (approximately half of all RDT-negative tests); (2) a RDT-negative result plus PCR test result (approximately half of all RDT-negative tests); or (3) RDT, PCR and culture results (all RDT-positive tests). Furthermore, as partial testing was not done completely at random (that is, our sampling scheme depended on the initial RDT result), we cannot assume a uniform prior for the probability of cholera. Thus, we accounted for the prior probability of test results conditional on the initial test result, cases (1) and (2) required marginalization and case (3) did not require marginalization because all three tests were performed (that is, the observation likelihood could be directly obtained through the multinomial likelihood detailed above).

For case (1), where only RDT-negative results were available, we let ${Y}_{1}$, ${Y}_{2}$ and ${Y}_{3}$ ∈ {0, 1} denote the binary random variables representing the results of RDT, PCR and culture, respectively. The prior probability (Pr) of a given PCR, ${Y}_{2}={y}_{2}$, and culture, ${Y}_{3}={y}_{3}$, test result given a negative RDT result, ${Y}_{1}=0$, is:

$$\Pr \left({Y}_{2}={y}_{2},{Y}_{3}={y}_{3}{\rm{|}}{Y}_{1}=0\right)=\frac{\Pr \left({Y}_{1}=0,{Y}_{2}={y}_{2},{Y}_{3}={y}_{3}\right)}{\Pr \left({Y}_{1}=0\right)}.$$

We obtained the numerator and denominators of the right-hand side of this equation above by integrating out the unobserved cholera infection status, ${x}$ ∈ {0, 1} as:

$$\begin{array}{ll}\Pr \left({Y}_{1}=0,{Y}_{2}={y}_{2},{Y}_{3}={y}_{3}\right)=\mathop{\sum}\limits _{x\in \left\{0,1\right\}}\Pr\Big({Y}_{1}=0,{Y}_{2}={y}_{2},{Y}_{3}={y}_{3}{\rm{|}}x\Big)\Pr \left(x\right)\\\qquad\qquad\qquad\qquad\qquad\qquad\;\;\, = \mathop{\sum}\limits_{x\in \left\{0,1\right\}}\Pr \Big({Y}_{1}=0{\rm{|}}{Y}_{2}={y}_{2},{Y}_{3}={y}_{3},x\Big)\\\qquad\qquad\qquad\qquad\qquad\qquad\;\;\quad\;\Pr \Big({Y}_{2}={y}_{2}{\rm{|}}{Y}_{3}={y}_{3},x\Big)\Pr \Big({Y}_{3}={y}_{3}{\rm{|}}x\Big)\Pr \left(x\right)\\\qquad\qquad\qquad\qquad\qquad\qquad\;\;\,= \mathop{\sum}\limits_{x\in \left\{0,1\right\}}\Pr \Big({Y}_{1}=0{\rm{|}}x\Big)\Pr \Big({Y}_{2}={y}_{2}{\rm{|}}x\Big)\\\qquad\qquad\qquad\qquad\qquad\qquad\;\;\quad\;\Pr \Big({Y}_{3}={y}_{3}{\rm{|}}x\Big)\Pr \left(x\right)\end{array}$$

where $\Pr \left(x\right)$ is the prior probability of cholera infection. The last equality was obtained by assuming that test results for a given sample are independent conditional on the participant’s infection status. We then used the above to complete the probability of the unobserved tests given a negative RDT test as follows:

$$\begin{array}{ll}\Pr \left({Y}_{2}={y}_{2},{Y}_{3}={y}_{3}{\rm{|}}{Y}_{1}=0\right) = \frac{\Pr \left({Y}_{1}=0,{Y}_{2}={y}_{2},{Y}_{3}={y}_{3}\right)}{\Pr \left({Y}_{1}=0\right)}\\\qquad\qquad\qquad\qquad\qquad\qquad\;\; = \frac{\sum _{x\in \left\{0,1\right\}}\Pr \left({Y}_{1}=0{\rm{|}}x\right)\Pr \left({Y}_{2}={y}_{2}{\rm{|}}x\right)\Pr \left({Y}_{3}={y}_{3}{\rm{|}}x\right)\Pr \left(x\right)}{\sum _{x\in \left\{0,1\right\}}\Pr \left({Y}_{1}=0{\rm{|}}x\right)\Pr \left(x\right)}\\\qquad\qquad\qquad\qquad\qquad\qquad\;\; = {\Phi }_{\{-,{y}_{2},{y}_{3}\}}\end{array}$$

where we used ${\Phi }_{\{-,{y}_{2},{y}_{3}\}}$ to denote the prior probability of PCR and culture results, ${y}_{2}$ and ${y}_{3},$ conditional on the negative RDT test result. The probabilities in the equation above were then expressed in terms of the test sensitivity, ${\theta }_{\mathrm{1,2,3}}^{+}$, and specificity, ${\theta }_{\mathrm{1,2,3}}^{-}$, as:

$$\begin{array}{ccc}\Pr \left({Y}_{i}=0{\rm{|}}x=0\right) & = & {\theta }_{i}^{-},\\ \Pr \left({Y}_{i}=1{\rm{|}}x=0\right) & = & 1-{\theta }_{i}^{-},\\ \Pr \left({Y}_{i}=0{\rm{|}}x=1\right) & = & {1-\theta }_{i}^{+},\\ \Pr \left({Y}_{i}=1{\rm{|}}x=1\right) & = & {\theta }_{i}^{+}.\end{array}$$

Priors on test performance were taken from an evaluation of the same RDT used in the present study, Cholkit, conducted in Dhaka Bangladesh^54^. Finally, the likelihood of a negative RDT result in case (1) given the probability vector ${{\mathbf{p}}}$ was obtained by marginalizing out the PCR and culture results accounting for their prior probabilities of occurrence:

$$\Pr \left(\{-,\bullet ,\bullet \}{\rm{|}}{\mathbf{p}}\right)=\sum _{{y}_{2}\in \left\{0,1\right\}{,y}_{3}\in \left\{0,1\right\}}{{\mathrm{multinomial}}}({{\mathbf{n}}}_{\{-,{y}_{2},{y}_{3}\}}{\rm{|}}{\mathbf{p}}){\Phi }_{\{-,{y}_{2},{y}_{3}\}},$$

where ${{\mathbf{n}}}_{\{-,{y}_{2},{y}_{3}\}}$ is the corresponding indicator vector for the outcome of interest.

For case (2), where RDT-negative and PCR test results were available, we considered the conditional probability for a given known PCR test result and marginalized out the unobserved culture result. We derived this case in a similar manner as:

$$\begin{array}{ll}\Pr \left({Y}_{3}={y}_{3}{{|}}{Y}_{1}=0,{Y}_{2}={y}_{2}\right) = \frac{\Pr \left({Y}_{1}=0,{Y}_{2}={y}_{2},{Y}_{3}={y}_{3}\right)}{\Pr \left({Y}_{1}=0,{Y}_{2}={y}_{2}\right)}\\\qquad\qquad\qquad\qquad\qquad\qquad\;\; = \frac{\sum _{x\in \left\{0,1\right\}}\Pr \left({Y}_{1}=0{{|}}x\right)\Pr \left({Y}_{2}={y}_{2}{{|}}x\right)\Pr \left({Y}_{3}={y}_{3}{{|}}x\right)\Pr \left(x\right)}{\sum _{x\in \left\{0,1\right\}}\Pr \left({Y}_{1}=0{{|}}x\right)\Pr \left({Y}_{2}={y}_{2}{{|}}x\right)\Pr \left(x\right)}\\\qquad\qquad\qquad\qquad\qquad\qquad\;\; = {{\Phi}^ {\prime}}_{\{-,{y}_{2},{y}_{3}\}}.\end{array}$$

We then used, for case (2):

$$\Pr \left(\{-,{y}_{2},\bullet \}{\rm{|}}{\mathbf{p}}\right)=\sum _{{y}_{3}\in \left\{0,1\right\}}{{\mathrm{multinomial}}}({n}_{\{-,{y}_{2},{y}_{3}\}}{\rm{|}}{\mathbf{p}}){{\Phi}^ {\prime} }_{\{-,{y}_{2},{y}_{3}\}}.$$

In the implementation of these prior probabilities, we set the prior probability of cholera to $\Pr \left(x\right)=0.2$.

Given the observed differences in AWD cases and RDT-positivity rates, we modeled age classes separately. We subdivided clinical samples into three age groups (<5, 5–64 and ≥65 years) and expanded the time-varying force of infection model to have age-specific cholera and non-cholera incidence. We also accounted for RDT batch effects caused by manufacturing error, which may have contributed to decreased test sensitivity, by separating the modeling period into two distinct periods: from the start of the study until 29 June 2021 and from 30 June 2021 until the end of the study period. The following priors were used in the cholera incidence model with no differences between age classes:

$$\begin{array}{ccc}{\alpha }_{{{\mathrm{chol}}}} & \sim & N\left(0,1\right),\\ {\alpha }_{-{{\mathrm{chol}}}} & \sim & N\left(0,1\right),\\ {{\mathrm{logit}}}\left({\theta }_{{{{\mathrm{RDT}}}}_{1}}^{+}\right) & \sim & N\left(0,0.75\right),\\ {{\mathrm{logit}}}\left({\theta }_{{{{\mathrm{RDT}}}}_{2}}^{+}\right) & \sim & N\left(3.89,0.95\right),\\ {{\mathrm{logit}}}\left({\theta }_{{{\mathrm{PCR}}}}^{+}\right) & \sim & N\left(1.04,0.34\right),\\ {{\mathrm{logit}}}\left({\theta }_{{{\mathrm{culture}}}}^{+}\right) & \sim & N\left(0.89,0.26\right),\\ {{\mathrm{logit}}}\left({\theta }_{{{{\mathrm{RDT}}}}_{1}}^{-}\right) & \sim & N\left(0,0.75\right),\\ {{\mathrm{logit}}}\left({\theta }_{{{{\mathrm{RDT}}}}_{2}}^{-}\right) & \sim & N\left(3.48,0.69\right),\\ {{\mathrm{logit}}}\left({\theta }_{{{\mathrm{PCR}}}}^{-}\right) & \sim & N\left(3.48,0.44\right),\\ {{\mathrm{logit}}}\left({\theta }_{{{\mathrm{culture}}}}^{-}\right) & \sim & N\left(6.9,0.55\right).\end{array}$$

**Extended Data Fig. 5:**
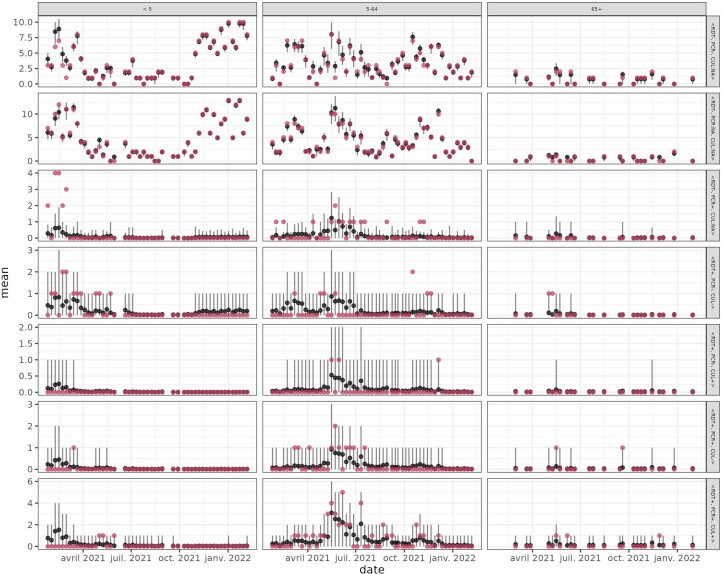
Posterior retrodictive checks of the clinical surveillance data. This plot illustrates the observed data (red dots) and the predicted mean values and 95% prediction intervals in black (*n* = 998).

**Extended Data Fig. 6:**
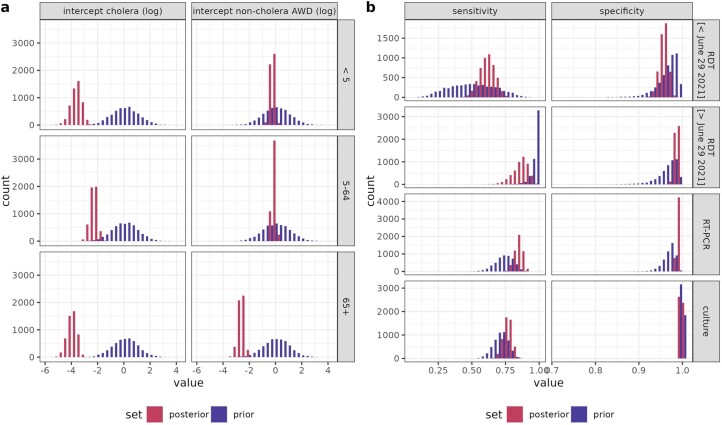
Clinical incidence model key parameter prior and posterior draws. Histograms of the intercepts of cholera and non-cholera AWD daily incidence (on log scale) (**a**) and the histograms of test sensitivity and specificity (**b**).

In addition, to see how well the model could reproduce testing data, we conducted posterior retrodictive checks (Extended Data Fig. 5 and the key posterior parameter draws observable in Extended Data Fig. 6). The overall result of this model component is the posterior distribution of the age-group-specific daily incidence of cholera and non-cholera AWD from study health facilities.

#### Seroincidence estimation framework

**Extended Data Fig. 7:**
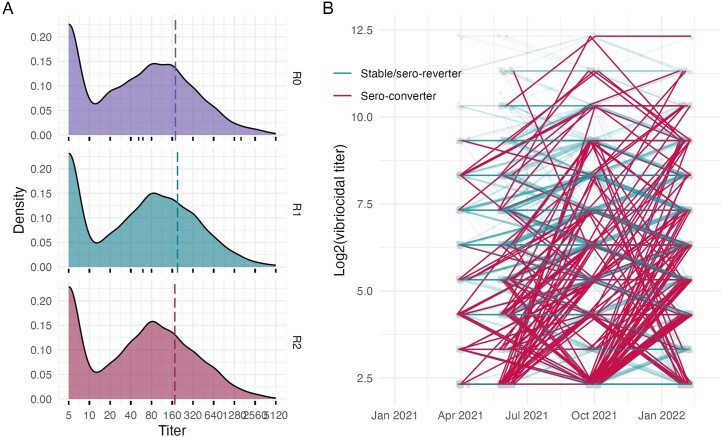
Vibriocidal results from the serosurvey. **a**. Density plot of *Vibrio cholerae* O1 Ogawa vibriocidal titers by serosurvey round where the dashed line indicates the population mean titer for the N = 1,785 individuals who provided serum samples for all three rounds of data collection. The rug plots below indicate the actual titer values measured. **b**. The trajectory of log2(vibriocidal titers) across serosurvey rounds grouped by those who seroconvert (≥2-fold rise in titer value across any two study visits) and those who remain stable or serorevert. The overall distribution of vibriocidal antibodies was similar across rounds of data collection, with 18.7% of participants having a titer ≥320 at baseline (during both enrollment periods; 17.6% during R0A and 19.1% during R0B), 21% during the first follow-up (R1), and 19.4% during the second follow-up (R2). Overall, 8.1% of participants had a ≥2-fold rise in vibriocidal titers across any two study visits including 14.9% among children 1–4 years old, 7.8%among those 5–64 years old and 8.6% among those 65 years and older. Four percent of individuals had a ≥2-fold rise in titers from enrollment to their first follow-up visit, and 4.2% had a ≥2-fold rise between the first follow-up and the final visit.

Our estimates of total infection incidence are based on serial measurements of vibriocidal antibodies developed in response to *V. cholerae* O1 Ogawa, the predominant regional serotype^55^, from each participant. Although simple definitions of seroincidence typically include (1) any rise in vibriocidal titer between visits or (2) any rise greater than a threshold (for example, twofold rise), these will be biased because titers decay quickly, often within months, and thus in a shorter time frame than our study visits (Extended Data Fig. 7). Also, these potential definitions do not account for measurement error in the vibriocidal assay. We, therefore, developed a Bayesian inference framework to estimate the seroincidence rate in the population based on serial measurements accounting for the decay kinetics of vibriocidal antibodies, the observed measurement error in the assay and the reconstructed epidemic curve of medically attended cholera cases as a proxy for the infection hazard experienced by study participants. We restricted these analyses to only those who participated in all three study visits (72% of the total enrolled). Participants of all three study visits were demographically similar to those lost to follow-up.

We assumed that postinfection antibody kinetics followed a previously published antibody kinetics model based on data from medically attended patients with cholera in Dhaka, Bangladesh^23^. In this model, vibriocidal antibodies were assumed to have an instantaneous boost after some delay postinfection from their baseline levels, and then to decay exponentially at a constant rate to a pre-exposure baseline level. The differences (or lack thereof) in vibriocidal measurement between rounds could indicate (1) absence of recent infection with any differences due to measurement error, (2) prior infection (before the last study visit) leading to a decay in vibriocidal titers or (3) an infection at some unknown time since the last visit, potentially with some decay in titer levels. Our inference framework estimates the likelihood of each of these possibilities for each participant, while utilizing the data from clinical surveillance to infer the likelihood of infections at each timepoint between measurements.

Specifically, to infer the probabilities of being infected at each serosurvey round, we marginalized over the possible infection dates before baseline and between each serosurvey round. The observation likelihood was composed of the titer observation likelihoods assuming the decay model, weighted by the probability of being infected in each period. This likelihood function was based on eight potential outcomes for individual-level infection status for the three periods corresponding to the three serological measurement rounds: the 6-month period preceding baseline and between each study round for each participant (for example,<0,0,0> indicates that the participant was not ‘recently’ infected before any of the study visits and <0,1,0> indicates that the participant was infected between the baseline and first visits).

For participant *i*, let ${\mu }_{i,T}$ be the probability of infection in a given time period *T* and *y*_*i,r*_ denote a serological measurement made at serosurvey round *r*. We denoted by *μ*_{0,0→1,1→2}_ and ${y}_{\{\mathrm{1,2,3}\}}$ the corresponding infection probabilities and serological measurements. We denoted by *x*(*t, τ*) the modeled vibriocidal titer measured at time *t* with exposure at a previous time *t* *−* *τ*, following the aforementioned exponential decay kinetic model. We let ${x}_{r}^{\star }(t)$ denote the modeled vibriocidal titer assuming that the baseline titer is equal to 0. Modeled antibody titer trajectories were linked to serological measurement through the probability density function ${f}_{{Y|X}}({\,y|x},\theta )$ with parameter $\theta$. We chose this observation model to be normally distributed (on the log scale of titers) with a known s.d. $\sigma$ (based on the variability of positive controls across plates within experiments done for the present study), which we denoted as ${f}_{N}({y|x})$ in the following. Finally, we denoted ${p}_{{t}_{{\mathrm{a}}}}^{{t}_{{\mathrm{b}}}}(\tau)$ as the prior conditional probability of infection at time *t*_a_ ≤ *τ* ≤ *t*_b_, given that infection did occur. With these notations we defined the eight possible sequences of infection statuses during each period:

$$\begin{array}{lll} < 0,0,0 > : & \left(1-{\mu }_{0}\right)\left(1-{\mu }_{0\to 1}\right)\left(1-{\mu }_{1\to 2}\right)\times \mathop{\prod }\limits_{r=1}^{3}\ {f}_{N}\left({y}_{i,r}|\gamma \right),\\ < 0,0,1 > : & \left(1-{\mu }_{0}\right)\left(1-{\mu }_{0\to 1}\right){\mu }_{1\to 2}\times \mathop{\prod }\limits_{r=1}^{2}\ {f}_{N}\left({y}_{i,r}|\gamma \right)\\&\times {\displaystyle{\int }}_{{t}_{2}}^{{t}_{3}}\ {f}_{N}\left({y}_{i,3}|x\left({t}_{3},\tau \right)\right){p}_{{t}_{2}}^{{t}_{3}}\left(\tau \right)d\tau ,\\ < 0,1,0 > : & \left(1-{\mu }_{0}\right){\mu }_{0\to 1}\left(1-{\mu }_{1\to 2}\right)\times {f}_{N}\left({y}_{i,1}|\gamma \right)\times {\displaystyle{\int }}_{{t}_{1}}^{{t}_{2}}\\&{f}_{N}\left({y}_{i,2}|x\left({t}_{2},\tau \right)\right){f}_{N}\left({y}_{i,3}|x\left({t}_{3},\tau \right)\right){p}_{{t}_{1}}^{{t}_{2}}\left(\tau \right)d\tau ,\\ < 0,1,1 > : & \left(1-{\mu }_{0}\right){\mu }_{0\to 1}{\mu }_{1\to 2}\times {f}_{N}\left({y}_{i,1}|\gamma \right)\\& \times {\displaystyle{\int }}_{{t}_{1}}^{{t}_{2}}{\displaystyle{\int }}_{{t}_{2}}^{{t}_{3}}\ {f}_{N}\left({y}_{i,2}|x\left({t}_{2},\tau \right)\right){f}_{N}\left({y}_{i,3}|x\left({t}_{3},\tau \right)\right.\\&\left.+{x}^{\star }({t}_{3},\tau {\prime} )\right){p}_{{t}_{1}}^{{t}_{2}}\left(\tau \right){p}_{{t}_{2}}^{{t}_{3}}\left(\tau {\prime} \right)d\tau d\tau {\prime} ,\\ < 1,0,0 > : & {\mu }_{0}\left(1-{\mu }_{0\to 1}\right)\left(1-{\mu }_{1\to 2}\right)\times \mathop{\prod }\limits_{r=1}^{3}{\displaystyle{\int }}_{{t}_{r-1}}^{{t}_{r}}\\&{f}_{N}\left({y}_{i,r}|x\left({t}_{r},\tau \right)\right){p}_{{t}_{r-1}}^{{t}_{r}}\left(\tau \right)d\tau ,\\ < 1,0,1 > : & {\mu }_{0}\left(1-{\mu }_{0\to 1}\right){\mu }_{1\to 2} \\& \times \mathop{\prod }\limits_{r=1}^{2}{\displaystyle{\int }}_{{t}_{0}}^{{t}_{1}}\ {f}_{N}\left({y}_{i,r}\left| x\left({t}_{r},\tau \right)\right.\right){p}_{{t}_{0}}^{{t}_{1}}\left(\tau \right)d\tau\\&\times{\displaystyle{\int }}_{{t}_{0}}^{{t}_{1}}{\displaystyle{\int }}_{{t}_{2}}^{{t}_{3}}\ {f}_{N}\left({y}_{i,2}|x\left({t}_{2},\tau \right)\right){f}_{N}\left({y}_{i,3}|x\left({t}_{3},\tau \right)\right.\\&\left.+{x}^{\star }({t}_{3},\tau {\prime} )\right){p}_{{t}_{0}}^{{t}_{1}}\left(\tau \right){p}_{{t}_{2}}^{{t}_{3}}\left(\tau {\prime} \right)d\tau d\tau {\prime} ,\\ < 1,1,0 > : & {\mu }_{0}{\mu }_{0\to 1}\left(1-{\mu }_{1\to 2}\right)\times {\displaystyle{\int }}_{{t}_{0}}^{{t}_{1}}\ {f}_{N}\left({y}_{i,1}|x\left({t}_{1},\tau \right)\right){p}_{{t}_{0}}^{{t}_{1}}\left(\tau \right)d\tau \\& \times {\displaystyle{\int }}_{{t}_{0}}^{{t}_{1}}{\displaystyle{\int }}_{{t}_{1}}^{{t}_{2}}\ {f}_{N}\left({y}_{i,2}|x\left({t}_{2},\tau \right)\right){f}_{N}\left({y}_{i,3}|x\left({t}_{3},\tau \right)\right.\\&\left.+{x}^{\star }({t}_{3},\tau {\prime} )\right){p}_{{t}_{0}}^{{t}_{1}}\left(\tau \right){p}_{{t}_{1}}^{{t}_{2}}\left(\tau {\prime} \right)d\tau d\tau {\prime} ,\\ < 1,1,1 > : & {\mu }_{0}{\mu }_{0\to 1}{\mu }_{1\to 2}\times {\displaystyle{\int }}_{{t}_{0}}^{{t}_{1}}\ {f}_{N}\left({y}_{i,1}|x\left({t}_{1},\tau \right)\right){p}_{{t}_{0}}^{{t}_{1}}\left(\tau \right)d\tau \times{\displaystyle{\int }}_{{t}_{0}}^{{t}_{1}}{\displaystyle{\int }}_{{t}_{1}}^{{t}_{2}}\\&{f}_{N}\left({y}_{i,2}|x\left({t}_{2},\tau \right)+{x}^{\star }({t}_{2},\tau {\prime} )\right){p}_{{t}_{0}}^{{t}_{1}}\left(\tau \right){p}_{{t}_{1}}^{{t}_{2}}\left(\tau {\prime} \right)d\tau d\tau {\prime} \\& \times{\displaystyle{\int }}_{{t}_{0}}^{{t}_{1}}{\displaystyle{\int }}_{{t}_{1}}^{{t}_{2}}{\displaystyle{\int }}_{{t}_{2}}^{{t}_{3}}\ {f}_{N}\left({y}_{i,3}|x\left({t}_{3},\tau \right)+{x}^{\star }({t}_{3},\tau {\prime} )+{x}^{\star }({t}_{3},\tau {\rm{\text{'}\text{'}}})\right)\\&{p}_{{t}_{0}}^{{t}_{1}}\left(\tau \right){p}_{{t}_{1}}^{{t}_{2}}\left(\tau {\prime} \right){p}_{{t}_{2}}^{{t}_{3}}\left(\tau {\rm{\text{'}\text{'}}}\right)d\tau d\tau {\prime} d\tau {\rm{\text{'}\text{'}}},\end{array}$$

where $\gamma$ is the baseline titer level in the kinetic model.

We then estimated the probability of infection, $\mu$, between any two arbitrary time points (*t*_l_ and *t*_k_) for a person, *i*, as a function of the infection hazard rate, assuming the time-varying incidence of medically attended cholera in the age class of participant *i* (*β*_*a*_) to be a valid proxy, scaled by an age-specific constant factor (*λ*_*a*_) as follows:

$${\mu }_{i}\left({t}_{{\mathrm{k}}},{t}_{{\mathrm{l}}}\right)=1-\exp \left(-\lambda_a {\int }_{{t}_{{\mathrm{k}}}}^{{t}_{{\mathrm{l}}}}\beta_{a} \left(\tau \right){\mathrm{d}}\tau \right).$$

In this formulation, we implicitly assumed that infections in the community overall are a function of the symptomatic cholera incidence leading to observed medically attended cases in the clinics. As we saw, boosts in antibodies continued during periods where there were few cholera cases detected at clinics. We therefore also considered a second model where seroincidence was a function of two components: the force of infection proportional to inferred symptomatic community infections and a constant force of infection independent of cases in the clinics. Expanding the model above, we assumed the existence of a time constant, antibody boost-causing source of infection independent of the time-varying hazard rate of cholera infection. We denoted the constant hazard rate as *λ*′ and estimated the probability of infection *μ* as:

$$\begin{array}{ccc}{\mu }_{i}\left({t}_{{\mathrm{k}}},{t}_{{\mathrm{l}}}\right) & = & 1-\exp \displaystyle\left(-\lambda_a {\int }_{{t}_{{\mathrm{k}}}}^{{t}_{{\mathrm{l}}}}\beta_a \left(\tau \right){\mathrm{d}}\tau -{\int }_{{t}_{{\mathrm{k}}}}^{{t}_{{\mathrm{l}}}}{\lambda }^{{\prime}}_a {\mathrm{d}}\tau \,\right)\\ & = & 1-\exp \displaystyle\left(-\lambda_a {\int }_{{t}_{{\mathrm{k}}}}^{{t}_{{\mathrm{l}}}}\beta_a \left(\tau \right){\mathrm{d}}\tau -{\lambda_a }^{{\prime} }({t}_{{\mathrm{l}}}-{t}_{{\mathrm{k}}})\right).\end{array}$$

To quantify the relative importance of the time-varying versus constant force of infection, we computed the ratio of marginal effects (Supplementary Table 7). For a given time period $[{t}_{{\mathrm{k}}},{t}_{{\mathrm{l}}}]$, letting $x=\lambda_{a} {\int }_{{t}_{{\mathrm{k}}}}^{{t}_{{\mathrm{l}}}}\beta_{a} \left(\tau \right){\mathrm{d}}\tau$ and *y* = *λ*_*a*_′(*t*_l_ *−* *t*_k_), the ratio of marginal effects was given by:

$$\begin{array}{ccc}R\left({t}_{{\mathrm{k}}},{t}_{{\mathrm{l}}}\right) & = & \frac{\frac{{\partial \mu }_{i}\left({t}_{{\mathrm{k}}},{t}_{{\mathrm{l}}}\right)}{\partial x}}{\frac{{\partial \mu }_{i}\left({t}_{{\mathrm{k}}},{t}_{{\mathrm{l}}}\right)}{\partial y}}\\ & = & \frac{{x{\mathrm{exp}}}\left(-\left(x+y\right)\right)}{{y{\mathrm{exp}}}\left(-\left(x+y\right)\right)}\\ & = & \frac{x}{y}=\frac{\lambda_a {\int }_{{t}_{{\mathrm{k}}}}^{{t}_{{\mathrm{l}}}}\beta_a \left(\tau \right){\mathrm{d}}\tau }{{\lambda }^{{\prime} }_a({t}_{{\mathrm{l}}}-{t}_{{\mathrm{k}}})}.\end{array}$$

**Extended Data Fig. 8:**
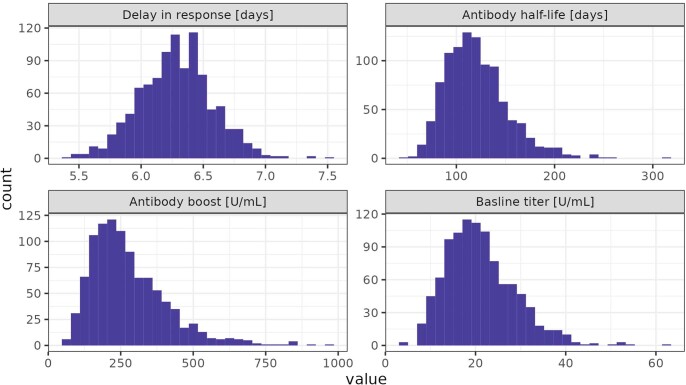
Posteriors of the antibody kinetic model. Posteriors of vibriocidal (Ogawa) antibody kinetics model parameters from Jones et al.^23^.The random draws from this posterior distribution are shown.

To account for uncertainty in the antibody kinetics model, we marginalized out kinetic model parameter, *θ*, using samples from the posterior distribution in ref. ^23^. The probability of a given sequence of infections Pr(<*l*, *m*, *n*> |*λ*) was therefore:

$$\begin{array}{ccc}\Pr \left( < l,m,n >|\lambda \right) & = &{\displaystyle{\int }}_{\Omega }^{.}\Pr \left( < l,m,n >|\lambda ,\Theta \right){\mathrm{d}}\theta \\ & \approx & \frac{1}{J}\mathop{\sum }\limits_{j=1}^{J}\Pr \left( < l,m,n >|\lambda ,{\Theta }^{\,j}\right)\\ & & {\Theta }^{\,j}\sim f\left(\Theta \right),\end{array}$$

where *f*(Θ) is the posterior distribution of parameter set Θ and ${\Theta }^{\,j}$ is a random draw from such a distribution. Random draws from this posterior distribution are shown in Extended Data Fig. 8.

Our final seroincidence model inferred the time-varying and constant seroincidence parameters jointly across the three age classes. We implemented pooling of both parameter values across age classes as:

$$\begin{array}{ccc}\log {\lambda }_{a} & \sim & \left({\mu }_{\log \lambda },{\sigma }_{\log \lambda }\right),\\ \log {{\lambda }^{{\prime} }}_{a} & \sim & \left({\mu }_{\log \lambda {\prime} },{\sigma }_{\log \lambda {\prime} }\right),\end{array}$$

where *μ* and *σ* are the mean and the s.d., respectively, of the time-varying and constant parameters on the log scale. We used the following priors in the final pooled seroincidence model:

$$\begin{array}{ccc}{\mu }_{\log \lambda } & \sim & N\left(-4,1\right),\\ {\sigma }_{\log \lambda } & \sim & N\left(0,1\right),\\ {\mu }_{\log \lambda {\prime} } & \sim & N\left(-5,1\right),\\ {\sigma }_{\log \lambda {\prime} } & \sim & N\left(0,1\right).\end{array}$$

**Extended Data Fig. 9:**
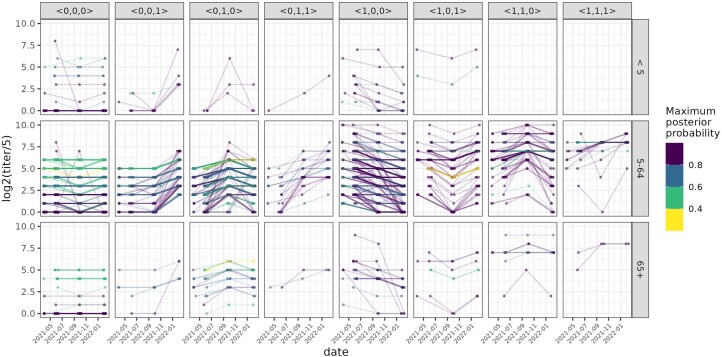
Posterior retrodictive checks of serological trajectories. This plot illustrates the titer trajectories for each participant classified by their most probable infection profile (panel/facet) along with the posterior probability of having that infection profile (color).

**Extended Data Fig. 10:**
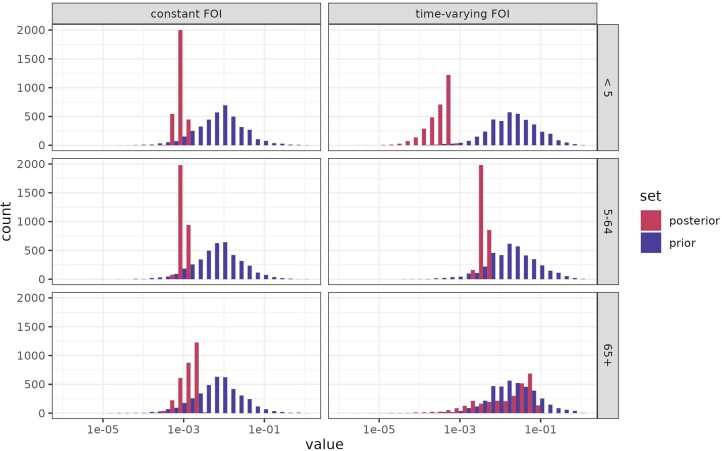
Seroincidence model key parameter prior and posterior draws. The key parameter prior and posterior draws from the seroincidence model by model type (assuming a constant infection hazard rate versus time-varying infection hazard rate) and age group.

We performed posterior retrodictive checks of the serological trajectories by examining the titer trajectories for each participant classified by their most probable infection profile along with the posterior probability of having that infection profile (Extended Data Fig. 9). The key parameter prior and posterior draws can be found in Extended Data Fig. 10.

Ultimately, we linked the cumulative medically attended cholera cases to cumulative seroincidence through the combined probabilities of symptomatic infection given infection, $\eta_a$, and the probability of health seeking given symptomatic infection by age class *a* (${\delta }_{a}$) as:

$$\mathop{\sum }\limits_{{t}_{{\mathrm{k}}}}^{{t}_{{\mathrm{l}}}}\beta _{a}(t)=P_{a}\times{\mu }_{a}\left({t}_{{\mathrm{k}}},{t}_{{\mathrm{l}}}\right)\times \eta_a \times {\delta}_{a},$$

where *P*_*a*_ is the total population in age class *a*. We inferred cumulative cases in the first inference step (Extended Data Fig. 4), cumulative seroincidence in the second and estimated posterior estimates from our health-seeking behavior survey; thus, the remaining unknown that we estimated was the probability of symptomatic infection.

We fitted these models using a Hamiltonian Monte Carlo (HMC) algorithm as implemented in Stan and assessed model convergence through visual inspection of trace plots and use of the Rhat statistics. We performed several posterior predictive checks to understand model fit and compared model formulations using an estimate of the LOO croosvalidation performance^56^.

#### Ethics approval

The study protocol was approved by the institutional review board (IRB) of the International Centre for Diarrheal Disease Research, Bangladesh (icddr,b), which includes the Research Review Committee and Ethical Review Committee, and the IRB of Johns Hopkins University. Informed written consent was taken from the participants (aged ≥18 years) and from the legal guardians of those aged <18 years.

#### Ethics and inclusion statement

We also endorsed the Nature Portfolio journal’s guidance on low-and-middle-income country (LMIC) authorship and inclusion. Data collected in the present study included local researchers from icddr,b and the participating health facilities in Chattogram, throughout the research process, from study design, implementation, data ownership to authorship. The conceptualization of the study was based on locally relevant needs in Bangladesh and gaps in our collective global understanding of cholera transmission and burden. Cholera is a disease that primarily affects individuals in LMICs and researchers at icddr,b have been at the forefront of cholera research for over half a century, notably contributing to the literature, which has been taken into account in our citations. The roles and responsibilities in both the study and the development of this manuscript were jointly agreed on between members of the participating institutions.

### Reporting summary

Further information on research design is available in the Nature Portfolio Reporting Summary linked to this article.

## Online content

Any methods, additional references, Nature Portfolio reporting summaries, source data, extended data, supplementary information, acknowledgements, peer review information; details of author contributions and competing interests; and statements of data and code availability are available at 10.1038/s41591-024-02810-4.

### Supplementary information

Supplementary Information Supplementary Tables 1–7.

Reporting Summary

## Data Availability

The deidentified data used in the analysis are available at github.com/HopkinsIDD/cholera_burden_cascade.

## Code availability

The code used to conduct analyses is available at github.com/HopkinsIDD/cholera_burden_cascade.
