# Author Correction: Clinical surveillance systems obscure the true cholera infection burden in an endemic region

**DOI:** 10.1038/s41591-024-03177-2

**Published:** 2024-07-09

**Authors:** Sonia T. Hegde, Ashraful Islam Khan, Javier Perez-Saez, Ishtiakul Islam Khan, Juan Dent Hulse, Md Taufiqul Islam, Zahid Hasan Khan, Shakeel Ahmed, Taner Bertuna, Mamunur Rashid, Rumana Rashid, Md Zakir Hossain, Tahmina Shirin, Kirsten E. Wiens, Emily S. Gurley, Taufiqur Rahman Bhuiyan, Firdausi Qadri, Andrew S. Azman

**Affiliations:** 1 Department of Epidemiology, Johns Hopkins Bloomberg School of Public Health, Baltimore, MA USA; 2 Infectious Disease Division, icddr,b (International Centre for Diarrhoeal Disease Research, Bangladesh), Dhaka, Bangladesh; 3 Unit of Population Epidemiology, Geneva University Hospitals, Geneva, Switzerland; 4 Geneva Centre for Emerging Viral Diseases, Geneva University Hospitals, Geneva, Switzerland; 5 Bangladesh Institute of Tropical and Infectious Diseases, Chattogram, Bangladesh; 6 Institute of Epidemiology, Disease Control and Research, Dhaka, Bangladesh; 7 Department of Epidemiology, Temple University, Philadelphia, PA USA; 8 Division of Tropical and Humanitarian Medicine, Geneva University Hospitals, Geneva, Switzerland

**Keywords:** Bacterial infection, Epidemiology

Correction to: *Nature Medicine* 10.1038/s41591-024-02810-4, published online 20 February 2024.

In the version of the article initially published, National Institutes of Health grant K22AI168389 was missing from the Acknowledgements and has now been added to the HTML and PDF versions of the article.

