# Supplementary material for: Clinical surveillance systems obscure the true cholera infection burden in an endemic region
Source: Nat Med. 2024 Feb 20;30(3):888–95. doi: 10.1038/s41591-024-02810-4 (PMC10957480; doi:10.1038/s41591-024-02810-4)
Supplement: Supplementary file 1 — Supplementary Tables 1–7. [file 41591_2024_2810_MOESM1_ESM.pdf]

nature medicine

Article https://doi.org/10.1038/s41591-024-02810-4

Clinical surveillance systems obscure the true cholera infection burden in an endemic region

In the format provided by the authors and unedited

Supplementary Tables

Supplementary Table 1: Medically-attended suspected case to true cholera case ratio. The ratio of suspected cases to the estimated true cholera incidence at health facilities decreases with age and is 10.5 (7.0-10.5) overall; for every 10.5 suspected cases that visit a health facility, 1 will be a true cholera case.

Age group	Suspected case incidence per 1,000	True cholera incidence per 1,000	Ratio
1-4	10.5	0.2 (0.1-0.3)	52.5 (35.0-105.0)
5-64	1.2	0.1 (0.1-0.2)	12.0 (6.0-12.0)
65+	1.9	0.5 (0.3-0.8)	3.8 (2.4-6.3)
Overall	2.1	0.2 (0.1-0.2)	10.5 (10.5-21.0)

Supplementary Table 2: Proportion of survey participants reporting that they would seek care for different diarrhea severities at one of the two official diarrhea treatment centres in the catchment area (Sitakunda Upazila Health Complex or BITID). There were no significant differences in healthcare seeking for moderate diarrhea across age groups.

Age group	N	Seek health='yes'	Proportion [mean (95% CrI)]
**Mild diarrhea**			
< 5	111	22	0.20 (0.13-0.28)
$5 - 64$	2221	330	0.15 (0.13-0.16)
65+	148	16	0.11 (0.07-0.17)
**Moderate diarrhea**			
< 5	111	29	0.27 (0.19-0.35)
$5 - 64$	2222	576	0.26 (0.24-0.28)
65+	148	36	0.25 (0.18-0.32)
**Severe diarrhea**			
< 5	111	54	0.49 (0.40-0.58)
$5 - 64$	2222	966	0.43 (0.41-0.46)
65+	148	63	0.43 (0.35-0.51)

Supplementary Table 3: Proportion of survey participants reporting that they would seek care for different diarrhea severities by age group and healthcare facility type in the catchment area. *Sitakunda facilities* refers to those individuals that would seek care at either the Sitakunda Upazila Health Complex or BITID, and *other facilities* refers to those individuals that would seek care at one of six other types of healthcare facilities in the study region: private hospitals, Chittagong General Hospital, CMCH college, Hathazari Upazila Health Complex, traditional healer, Union health office. Participants were less likely to seek care at a pharmacy with increased disease severity.

Age group	Prop. seeking any care (n/N)	Prop. visiting Sitakunda facilities (n/N)	Prop. visiting pharmacies (n/N)	Prop. visiting other facilities (n/N)
**Mild diarrhea**				
1-4	0.81 (90/111)	0.20 (22/111)	0.59 (66/111)	0.02 (2/111)
5-64	0.75 (1669/2220)	0.15 (330/2220)	0.58 (1293/2220)	0.02 (46/2220)
65+	0.76 (113/148)	0.11 (16/148)	0.64 (95/148)	0.01 (2/148)
Overall	0.76 (1872/2479)	0.15 (368/2479)	0.59 (1454/2479)	0.02 (50/2479)
**Moderate diarrhea**				
1-4	0.92 (102/111)	0.26 (29/111)	0.58 (64/111)	0.08 (9/111)
5-64	0.86 (1901/2222)	0.26 (576/2222)	0.49 (1090/2222)	0.11 (235/2222)
65+	0.84 (125/148)	0.24 (36/148)	0.49 (73/148)	0.11 (16/148)
Overall	0.86 (2128/2481)	0.26 (641/2481)	0.49 (1227/2481)	0.10 (260/2481)
**Severe diarrhea**				
1-4	0.95 (106/111)	0.49 (54/111)	0.41 (45/111)	0.06 (7/111)
5-64	0.94 (2079/2222)	0.43 (966/2222)	0.40 (888/2222)	0.10 (225/2222)
65+	0.96 (142/148)	0.43 (63/148)	0.40 (59/148)	0.14 (20/148)
Overall	0.94 (2327/2481)	0.44 (1083/2481)	0.40 (992/2481)	0.10 (252/2481)

Supplementary Table 4: Estimates of annualized suspected, true cases, and infections by age group visiting healthcare facilities and in the community. The total population for each age group and overall is shown in the first row.

Estimate	1-4 years (N)	5-64 years (N)	65+ years (N)	Overall (N)
Total population	42,095	389,994	18,006	450,095
**Healthcare facility**				
Suspected cholera cases	442	470	35	947
RDT positive suspected cases	15	60	6.6	82
True cholera cases (clinics)	7.9 (4.1-13)	58 (46-71)	8.9 (4.8-14)	74 (61-89)
**Community**				
True cholera cases (community + clinics)	30 (15-53)	222 (175-276)	37 (19-61)	290 (235-352)
Infections	13,714 (9,870-17,454)	210,354 (202,065-219,199)	10,848 (9,232-12,354)	240,643 (231,502-250,100)

Supplementary Table 5: Number of individuals using different drinking water sources in the one week prior to the survey among the serological cohort (N=1,785; multiple sources allowed per individual). Notably, the use of piped and tap water as the primary water source in the week prior to the survey markedly reduced across seasons, from spring to winter, within the course of one year. Despite multiple water sources being permitted to be selected per individual, there was more piped and tap water in use during the high transmission season, which could be indicative of a lack of consistent water service and/or contamination.

Serosurvey round	R1	R2	R3
Season	Spring	Fall	Winter
Protected well (n)	3	0	0
Unprotected spring (n)	3	0	0
Rainwater (n)	0	0	3
Piped (n)	254	112	86
Tap (n)	135	1	18
Surface (n)	0	0	101
Tubewell (n)	1403	1683	1685

Supplementary Table 6: Estimates of true annualized symptomatic incidence rates of *V. cholerae* per 1,000 population by diarrhea severity. These data show that the true symptomatic incidence rate (using data from the community and clinics) vary only slightly across the different definitions of diarrhea (mild, moderate, severe), which determine the probability of healthcare seeking. As the severity of diarrhea increases, our estimates of the true incidence of symptomatic cholera decreases.

Diarrhea type	1-4	5-64	65+	Overall
Mild	0.9 (0.4-1.7)	1.0 (0.8-1.2)	4.6 (2.1-8.2)	1.1 (0.9-1.4)
Moderate	0.7 (0.4-1.2)	0.6 (0.4-0.7)	2.0 (1.0-3.4)	0.6 (0.5-0.8)
Severe	0.4 (0.2-0.6)	0.3 (0.3-0.4)	1.2 (0.6-1.9)	0.4 (0.3-0.5)

Supplementary Table 7: Ratio of the marginal effects to quantify the relative importance of the time-vraying versus constance force of infection seroincidence models.

Age categories	Marginal effect (CI)
1-4	0.17 (0.01-0.79)
5-64	0.90 (0.12-3.78)
65+	0.65 (0.02-2.78)
