# Supplementary material for: Clinical surveillance systems obscure the true cholera infection burden in an endemic region
Source: Nat Med. 2024 Feb 20;30(3):888–95. doi: 10.1038/s41591-024-02810-4 (PMC10957480; doi:10.1038/s41591-024-02810-4)
Supplement: Supplementary file 2 — Reporting Summary [file 41591_2024_2810_MOESM2_ESM.pdf]

Corresponding author(s): Andrew Azman
Last updated by author(s): 18/12/23

Reporting Summary

Nature Portfolio wishes to improve the reproducibility of the work that we publish. This form provides structure for consistency and transparency in reporting. For further information on Nature Portfolio policies, see our Editorial Policies and the Editorial Policy Checklist.

Statistics

For all statistical analyses, confirm that the following items are present in the figure legend, table legend, main text, or Methods section.

n/a	Confirmed	
☐	☒	The exact sample size (*n*) for each experimental group/condition, given as a discrete number and unit of measurement
☐	☒	A statement on whether measurements were taken from distinct samples or whether the same sample was measured repeatedly
☒	☐	The statistical test(s) used AND whether they are one- or two-sided<br>*Only common tests should be described solely by name; describe more complex techniques in the Methods section.*
☐	☒	A description of all covariates tested
☐	☒	A description of any assumptions or corrections, such as tests of normality and adjustment for multiple comparisons
☐	☒	A full description of the statistical parameters including central tendency (e.g. means) or other basic estimates (e.g. regression coefficient) AND variation (e.g. standard deviation) or associated estimates of uncertainty (e.g. confidence intervals)
☒	☐	For null hypothesis testing, the test statistic (e.g. *F*, *t*, *r*) with confidence intervals, effect sizes, degrees of freedom and *P* value noted<br>*Give P values as exact values whenever suitable.*
☐	☒	For Bayesian analysis, information on the choice of priors and Markov chain Monte Carlo settings
☐	☒	For hierarchical and complex designs, identification of the appropriate level for tests and full reporting of outcomes
☐	☒	Estimates of effect sizes (e.g. Cohen's *d*, Pearson's *r*), indicating how they were calculated

Our web collection on statistics for biologists contains articles on many of the points above.

Software and code

Policy information about availability of computer code

Data collection	RedCap (verson 13.1.3) software and ODK Collect/ODK Central were used to collect questionnaire data from study participants in clinical surveillance and the serosurvey/healthcare seeking survey, respectively.
Data analysis	The code used to conduct all analyses and generate the supplement are available at https://github.com/HopkinsIDD/cholera_burden_cascade

For manuscripts utilizing custom algorithms or software that are central to the research but not yet described in published literature, software must be made available to editors and reviewers. We strongly encourage code deposition in a community repository (e.g. GitHub). See the Nature Portfolio guidelines for submitting code & software for further information.

Data

Policy information about availability of data

All manuscripts must include a data availability statement. This statement should provide the following information, where applicable:

- Accession codes, unique identifiers, or web links for publicly available datasets
- A description of any restrictions on data availability
- For clinical datasets or third party data, please ensure that the statement adheres to our policy

The data used in the analysis will be made available upon publication at https://github.com/HopkinsIDD/cholera_burden_cascade.

Human research participants

Policy information about studies involving human research participants and Sex and Gender in Research.

Reporting on sex and gender	Only sex data was collected from study participants as the study was conducted in Bangladesh, where the sociological difference is less well understood, and our objective was to understand how to accurately estimate infection incidence of Vibrio cholerae. Historically data has shown little differences in disease incidence by sex, and any differences have been attributed to those individuals who work outside the household more and therefore are more likely to have an increased number of exposures. We report the descriptive differences in those who test positive for V. cholerae by sex in the study tables but do not conduct our more rigorous statistical analyses by sex (or gender) and rather report results disaggregated by age, which proved to be a more meaningful covariate.
Population characteristics	See the Behavioural & Social Sciences section below.
Recruitment	For clinical surveillance, we recruited participants from both the in-patient and out-patient wards of our study health facilities, and attempted to enroll all suspected cases ≥1 years old presenting with non-bloody, acute watery diarrhea (3 or more loose stools in the 24-hours preceding the visit). After obtaining informed consent we administered a short, structured questionnaire and collected a stool (or rectal swab) specimen for laboratory analyses. As we did not enroll many out-patients in clinical surveillance, our estimates of clinical incidence rely heavily on those experiencing more severe diarrhea-like symptoms who visited the in-patient wards.<br><br>For the serosurvey and healthcare seeking survey, we enrolled a population representative cohort in the study region, Sitakunda. To enroll households, we used two-stage sampling based on satellite imagery (Airbus, Pléiades 1B sensor) with digitized building footprints classified as single or multi-story units, where we first divided the Sitakunda subdistrict into 1km2 grid-cells and then randomly selected grid-cells proportional to the number of households in each with replacement. Within each selected grid-cell, we randomly selected structures (or GPS coordinates) weighted by whether they were classified as single- or multi-story units; the number of structures selected by grid-cell varied and only one structure was enrolled per GPS coordinate selected. If no structure was found at the point or the structure located at the point was not residential, the study team attempted to enroll the nearest residential household within 20 meters or proceeded to the next assigned point if no residential household existed within 20 meters. If the structure found was multi-story, the study team enumerated the number of residential households inside and generated a random number to determine which household to attempt to enroll. If no one was at the household once it was found, the study team attempted to revisit up to three times in the following 24 hours. If no one was at home after the attempted revisits or the household refused to participate, the study team proceeded to the next point (or attempted to enroll the next closest residence to the right if in a multi-story unit). After receiving verbal consent from the head of household (or representative), we attempted to enroll all persons ≥1 year of age that were members of the household (i.e., those who regularly sleep in the household and eat there) and asked for written consent (and assent for those 7-17 years old) from each person. Each enrolled household had two follow-up visits at approximately 4-month intervals, and at each follow-up visit, members of households who were not previously enrolled (or declined to participate in the previous round) were eligible to participate, and all consenting participants were administered a follow-up individual-level questionnaire, venous blood draw, and household heads a follow-up household-level questionnaire.<br><br>We do not think any substantial biases were incurred based on our recruitment process, as we enrolled all participants in the household except for those less than 1 year of age. As there was some in- and out-migration from households between visits, we experienced loss to follow-up. However, those who were lost to follow-up were not demographically different than those individuals who remained in our serological cohort. In clinical surveillance, we enrolled all suspected cases visiting the health facility and had zero refusals for participation.
Ethics oversight	The study protocol was approved by the Institutional Review Board (IRB) of the International Centre for Diarrheal Disease Research, Bangladesh (icddr,b) which includes Research Review Committee (RRC) and Ethical Review Committee (ERC) and of Johns Hopkins University.

Note that full information on the approval of the study protocol must also be provided in the manuscript.

Field-specific reporting

Please select the one below that is the best fit for your research. If you are not sure, read the appropriate sections before making your selection.

☐ Life sciences ☒ Behavioural & social sciences ☐ Ecological, evolutionary & environmental sciences

For a reference copy of the document with all sections, see nature.com/documents/nr-reporting-summary-flat.pdf

Behavioural & social sciences study design

All studies must disclose on these points even when the disclosure is negative.

Study description	We conducted a quantitative, enhanced surveillance study in Sitakunda, Bangladesh, which included a longitudinal serological survey,

Study description	a cross-sectional healthcare seeking survey, and daily clinical surveillance during the study period from January 24, 2021 through February 13, 2022.
Research sample	For clinical surveillance, our research sample included all suspected cases ≥1 year of age with non-bloody, acute watery diarrhea (3 or more loose stools in the 24-hours preceding the visit) that sought care at both the in-patient and out-patient wards of our study health facilities during the study period. For the serosurvey and healthcare seeking survey, our research sample was a population representative cohort of Sitakunda, a subdistrict in the district of Chattogram, Bangladesh. We attempted to enroll all persons ≥1 year of age that were members of the household (i.e., those who regularly sleep in the household and eat there).<br><br>We chose the Sitakunda subdistrict as our study site as it has been previously shown to have both moderate-to-high seroincidence and clinical risk. We restricted our study sample to those ≥1 year of age as it is difficult to acquire blood samples from such small children and most children <1 year of age are breastfed in this study population and therefore experience minimal cholera risk.
Sampling strategy	For clinical surveillance, we attempted to enroll all suspected cases (comprehensive sampling) that visited the study health facilities under surveillance during the study period. No sample size calculation was used to determine the number of clinical cases needed to be surveilled. For the serosurvey and healthcare seeking survey, we determined a minimum sample size of 1,632 individuals (~408 households) was needed to detect a significant change in seroincidence of V. cholerae between the first and third longitudinal serosurvey. Based on data from the previous 2015 nationally representative serosurvey conducted in Bangladesh, we powered this study to detect a 5% increase in V. cholerae seroincidence over the course of the cholera season assuming a baseline seroprevalence of 5% (i.e., a rise from 5% to 10% seroincidence rate over the 6-month cholera season). The baseline seroincidence rate indicates that 5% of the population had a meaningful immunologic exposure to V. cholerae in the previous 6 months of round 1. This led us to a sample size estimate of 1,632 individuals (~408 households) to detect a significant change in our seroincidence estimates from across these time points, assuming an intra-class correlation coefficient (ICC - the strength of correlation within households) for V. cholerae infection of 0.6, a design effect of 2.8 (assuming an average household size of 4), and 35% loss to follow-up between the first and the last survey rounds. For the serosurvey, we employed two-stage cluster sampling whereby we first divided the Sitakunda subdistrict into 1km2 grid-cells and then randomly selected grid-cells proportional to the number of households in each with replacement. Within each selected grid-cell, we randomly selected structures (or GPS coordinates) weighted by whether they were classified as single- or multi-story units.
Data collection	We used RedCap (Johns Hopkins RedCap server) to collect and store data for the clinical surveillance and used Open Data Kit (ODK) to collect and store data for the serologic and healthcare seeking surveys. All data was collected using tablets and stored on a secure server. Trained study staff (research assistants), who were aware of the study objectives, collected the survey data on tablets and medical technicians who were accompanied by community healthcare workers collected the serum samples within each household. Trained nurses or medical technicians, who were aware of the study objectives, collected the stool samples in each clinical health facility. All study staff were aware of the study objectives and hypotheses.
Timing	From January 24, 2021 through February 13, 2022, we surveilled the in-patient and out-patient wards of our study health facilities, and attempted to enroll all suspected cases ≥1 years old into clinical surveillance. Between 27-March-2021 and 13-June-2021, we enrolled a population representative cohort in Sitakunda, including a ~1-month gap due to a national COVID-19 related lockdown (referred to as rounds R0A & R0B throughout). Each enrolled household had two follow-up visits at approximately 4-month intervals (R1 from 21-September-2021 to 9-October-2021 and R2 from 25-January-2022 to 13-February-2021).
Data exclusions	We included all clinical surveillance participants within the indicated study period and all participants of the longitudinal serological cohort that completed all three study visits. Participants of the serosurvey that were lost to follow-up or were enrolled in later rounds of the study, were excluded from the analysis. We also included data from all participants that partook in the healthcare seeking survey, which occurred during the first round of the longitudinal serosurvey and among the same population.
Non-participation	In the first round of the serosurvey, 2,481 individuals were enrolled into the study, however, 1,785 individuals were retained and not lost to follow-up across all three rounds of the serosurvey. Individuals that were lost to follow-up did not have distinguishing demographic features. Reasons for being lost to follow-up included not being at the household at the time of the survey, marriage or moving outside the household. As the enrollment of the baseline cohort occurred during the height of the COVID-19 pandemic, individual migration patterns shifted significantly over the course of the study.
Randomization	There was no randomization in the selection of participants in clinical surveillance as we attempted to enroll all participants that visited the health facilities that met the suspected case definition, identified by the inpatient and/or outpatient register or logbook. To control for covariates in the analyses, we stratified analyses by age category.<br><br>To select households for enrollment into the longitudinal serosurvey and healthcare seeking survey, we used two-stage sampling based on satellite imagery with digitized building footprints classified as single or multi-story units, where we first divided the Sitakunda subdistrict into 1km2 grid-cells and then randomly selected grid-cells proportional to the number of households in each with replacement. Within each selected grid-cell, we randomly selected structures (or GPS coordinates) weighted by whether they were classified as single- or multi-story units; the number of structures selected by grid-cell varied and only one structure was enrolled per GPS coordinate selected. If no structure was found at the point or the structure located at the point was not residential, the study team attempted to enroll the nearest residential household within 20 meters or proceeded to the next assigned point if no residential household existed within 20 meters. If the structure found was multi-story, the study team enumerated the number of residential households inside and generated a random number to determine which household to attempt to enroll. If no one was at the household once it was found, the study team attempted to revisit up to three times in the following 24 hours. If no one was at home after the attempted revisits or the household refused to participate, the study team proceeded to the next point (or attempted to enroll the next closest residence to the right if in a multi-story unit). We attempted to enroll all participants ≥1 year of age in each household. To control for covariates in the analyses, we stratified analyses by age category.

Reporting for specific materials, systems and methods

We require information from authors about some types of materials, experimental systems and methods used in many studies. Here, indicate whether each material, system or method listed is relevant to your study. If you are not sure if a list item applies to your research, read the appropriate section before selecting a response.

Materials & experimental systems

n/a	Involved in the study
☒	☐ Antibodies
☒	☐ Eukaryotic cell lines
☒	☐ Palaeontology and archaeology
☒	☐ Animals and other organisms
☒	☐ Clinical data
☒	☐ Dual use research of concern

Methods

n/a	Involved in the study
☒	☐ ChIP-seq
☒	☐ Flow cytometry
☒	☐ MRI-based neuroimaging
